# Supplementary figures and images for: Cohesin-independent STAG proteins interact with RNA and R-loops and promote complex loading
Source: eLife. 2023 Apr 3;12:e79386. doi: 10.7554/eLife.79386 (PMC10238091; doi:10.7554/eLife.79386)

Figure 1c Source Data.

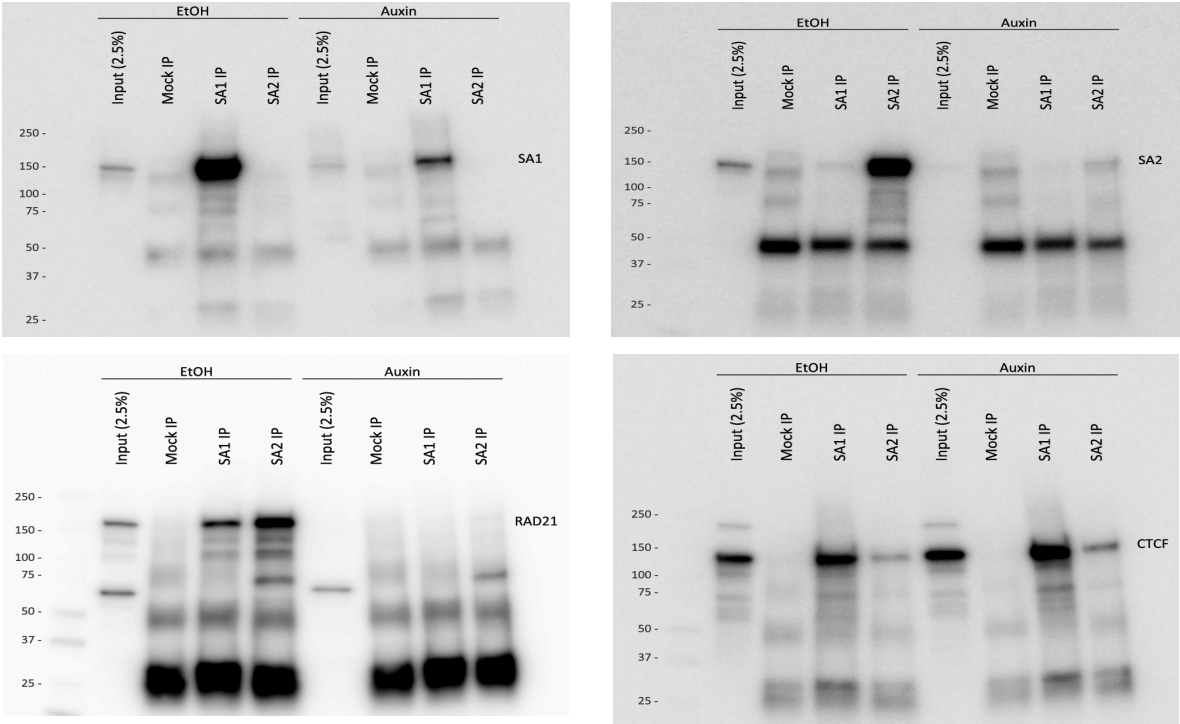

Figure 1d Source Data.

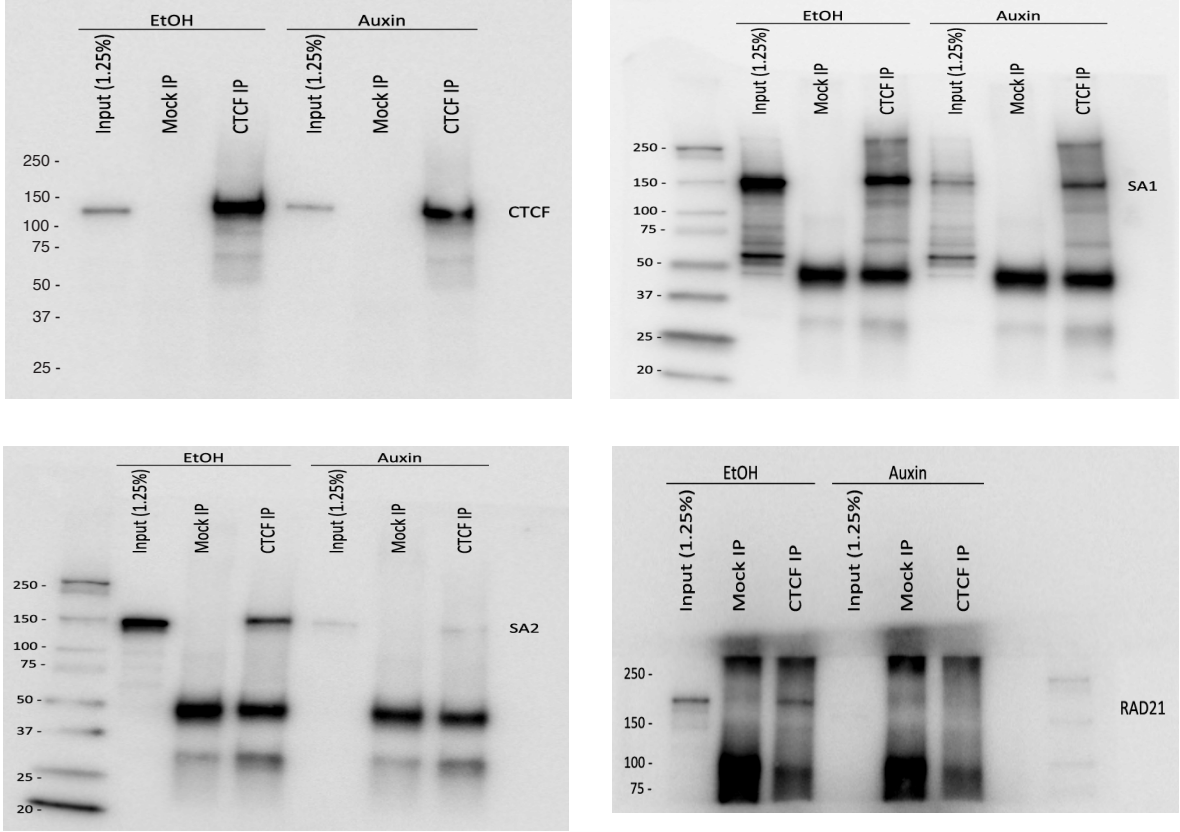

Supplement: Figure 1—source data 1. [file elife-79386-fig1-data1.zip › Figure 1 - source data 1/Source Data_Figure 1.pdf]

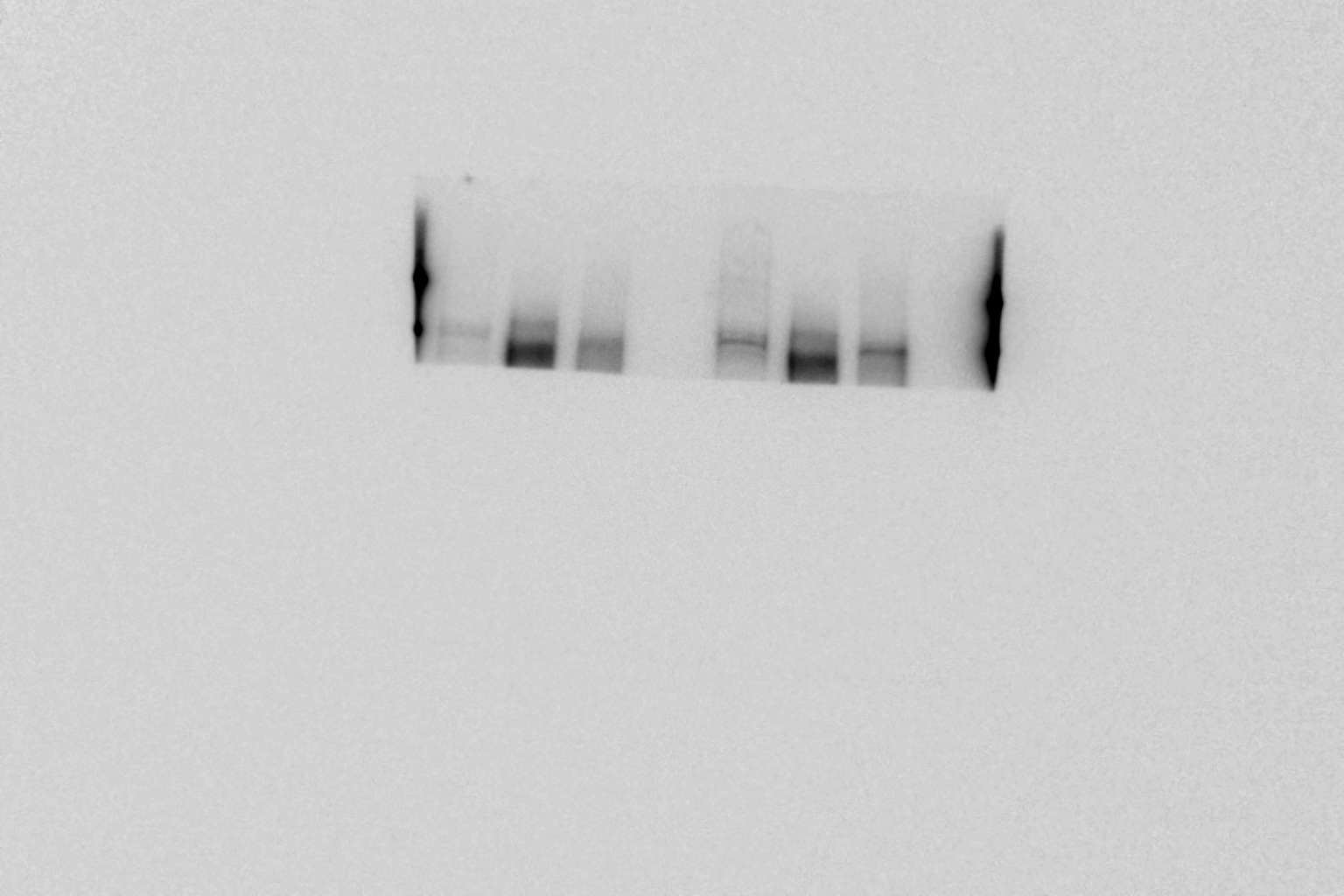

Supplement: Figure 2—source data 1. [file elife-79386-fig2-data1.zip › Figure 2 - source data 1/Figure 2d_source data 5.tif]

Figure 2a Source Data.

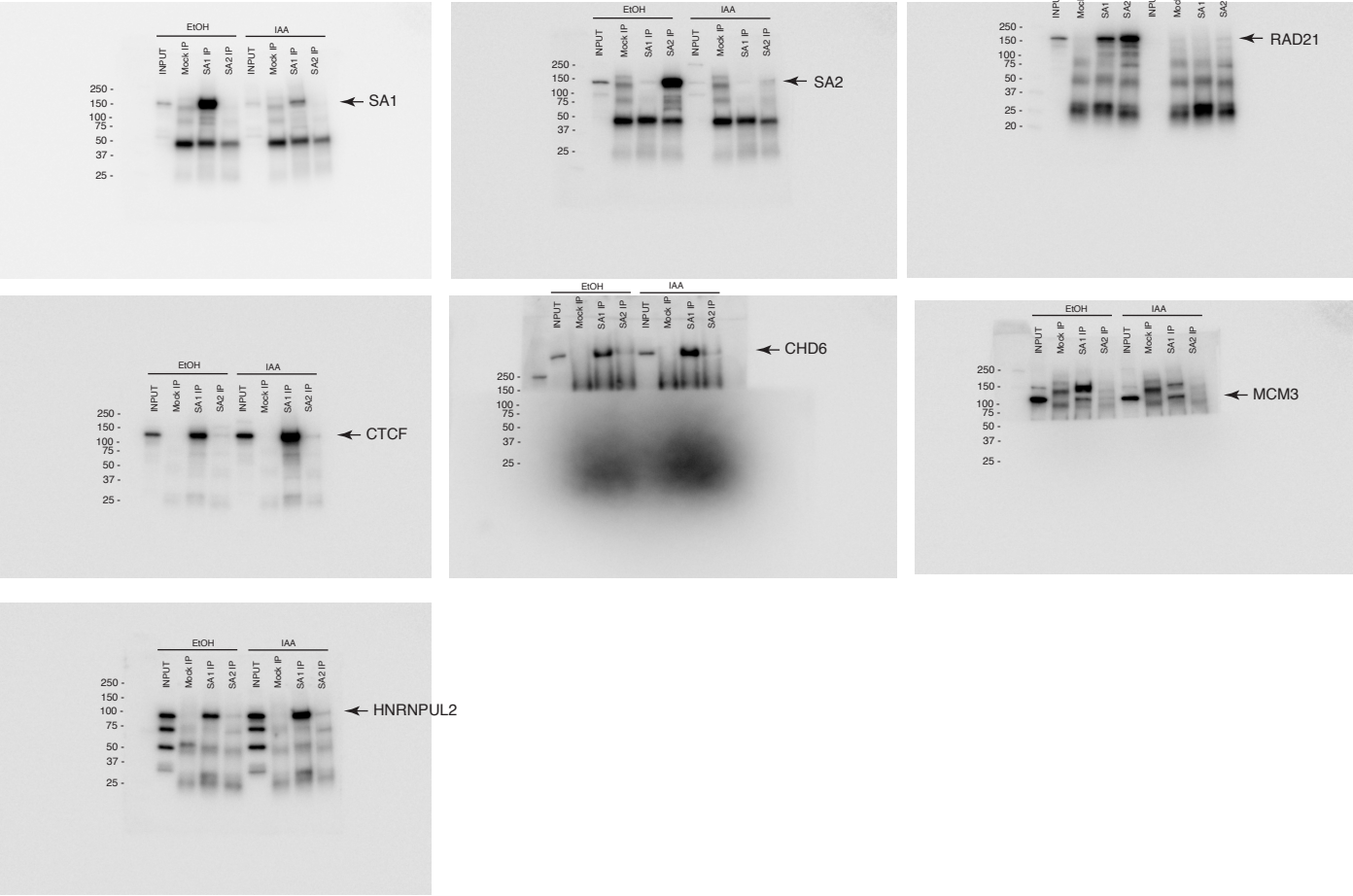

Figure 2d Source Data.

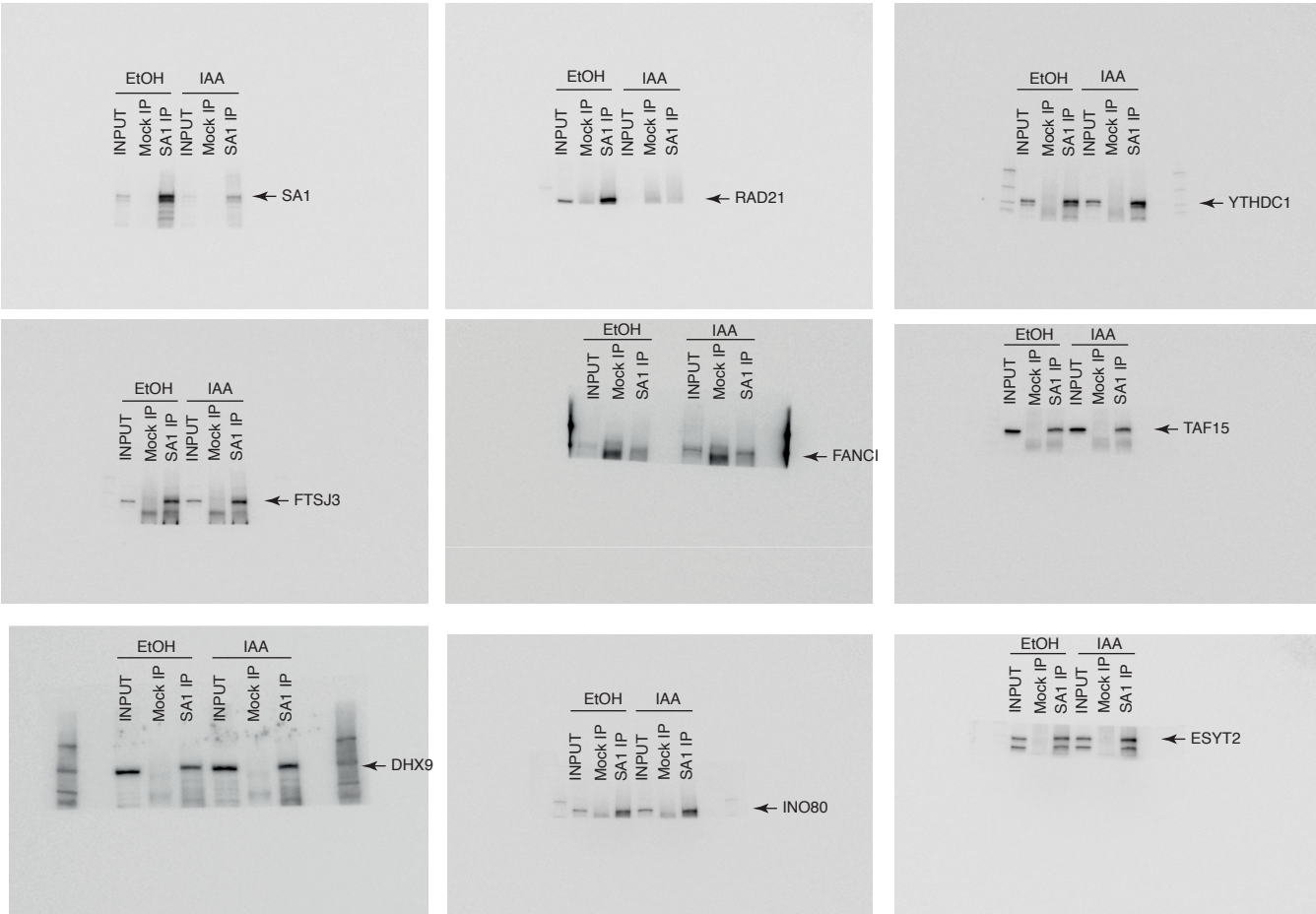

Supplement: Figure 2—source data 1. [file elife-79386-fig2-data1.zip › Figure 2 - source data 1/Source Data_Figure 2.pdf]

Figure S2b Source Data.

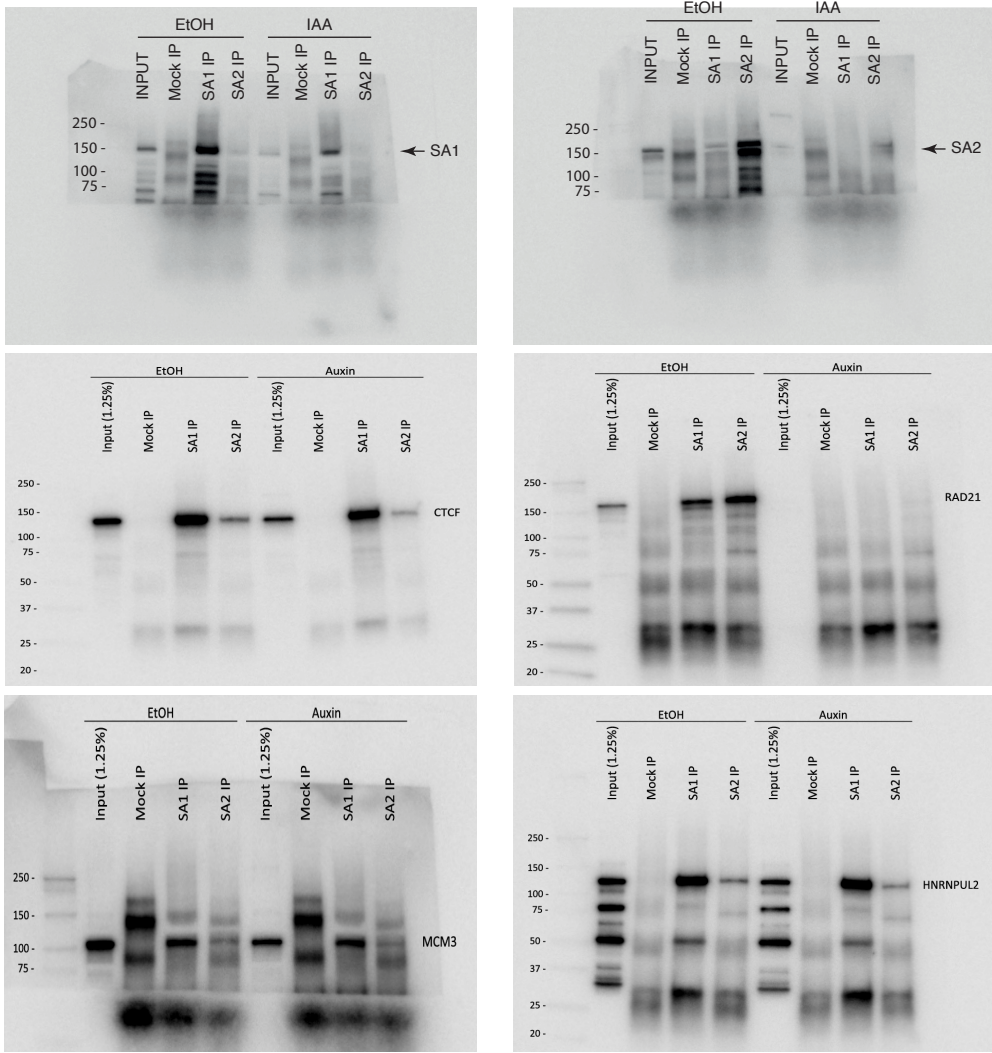

Supplement: Figure 2—figure supplement 1—source data 1. [file elife-79386-fig2-figsupp1-data1.zip › Figure 2 - figure supplement 1 - source data 1/Source Data_SUPP Figure 2.pdf]

Figure 3a Source Data.

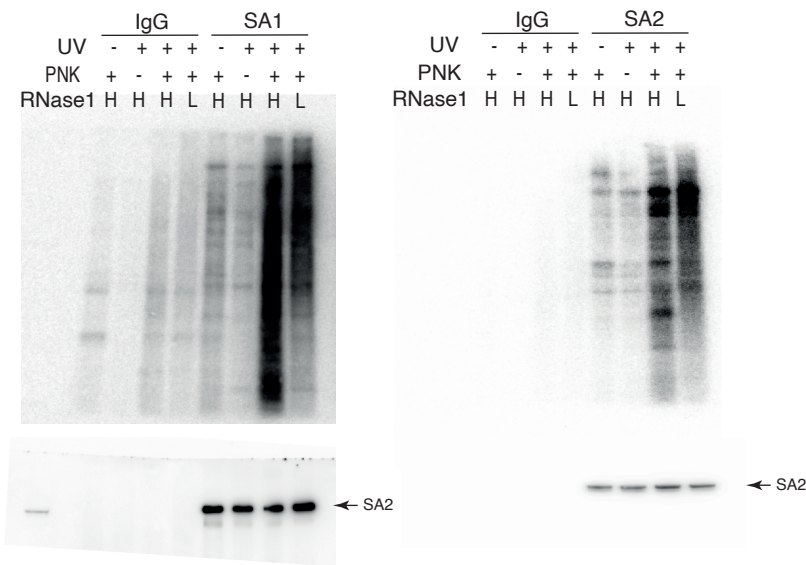

Figure 3b Source Data.

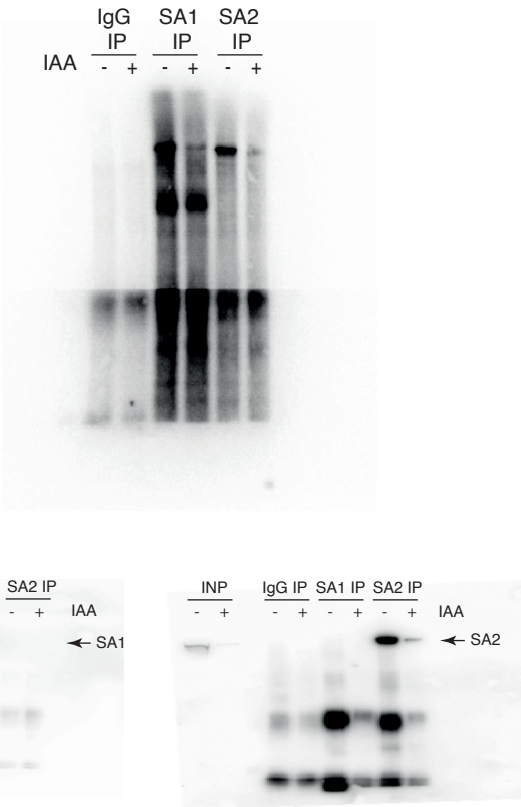

Figure 3g Source Data.

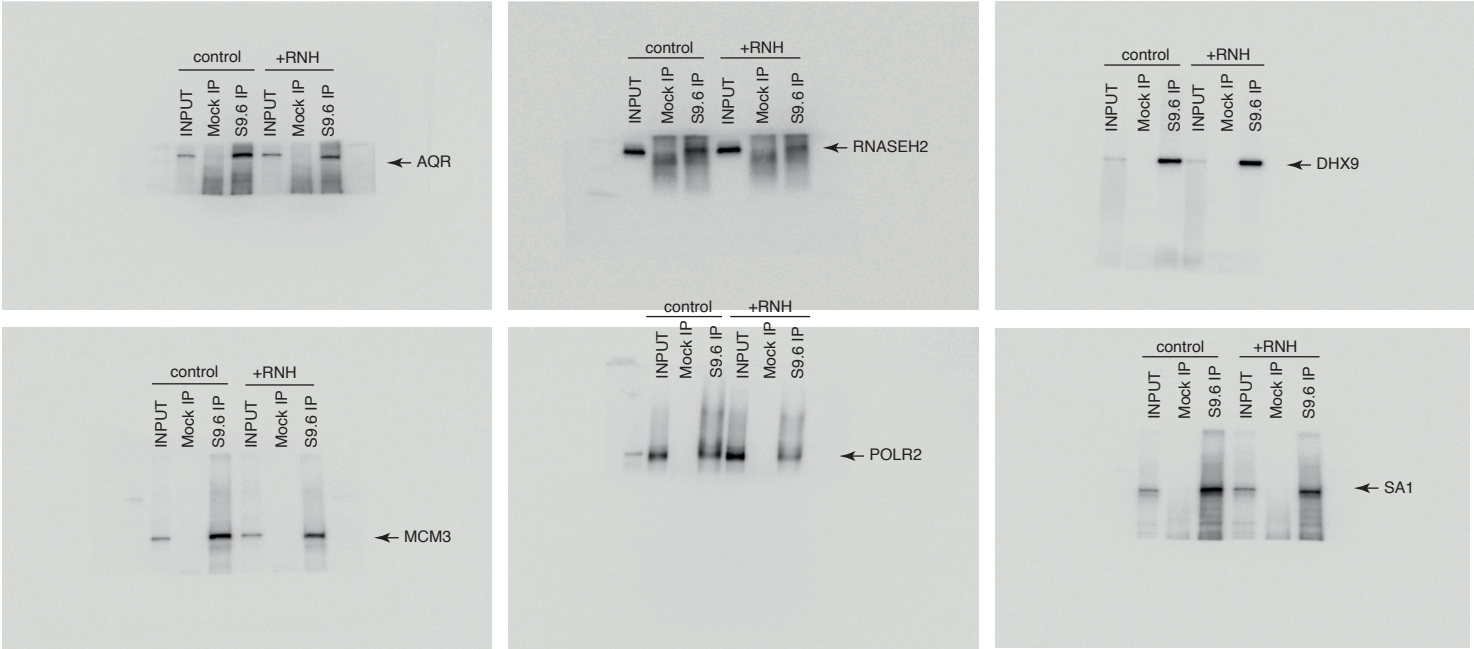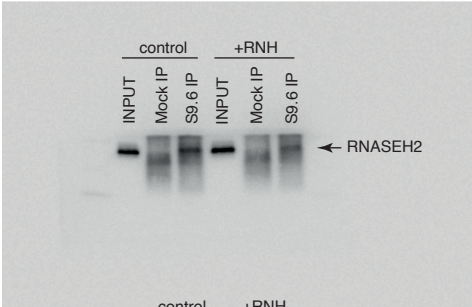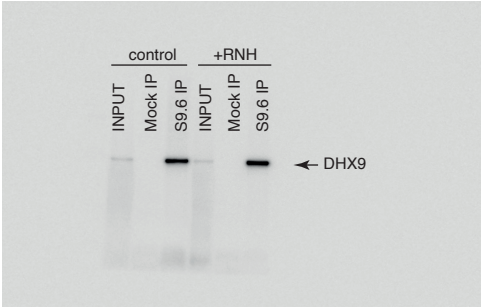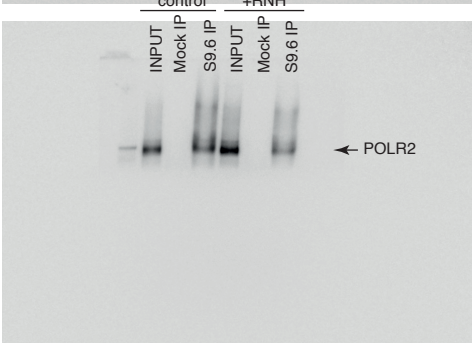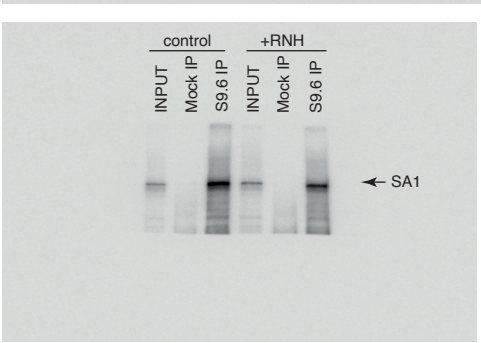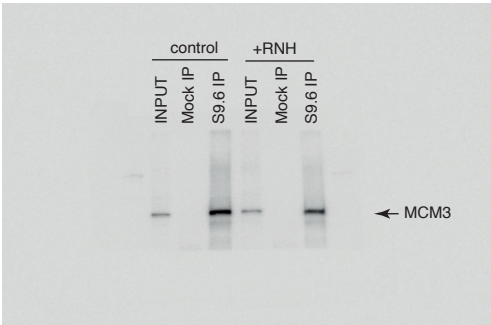

Supplement: Figure 3—source data 1. [file elife-79386-fig3-data1.zip › Figure 3 - source data 1/Source Data_Figure 3.pdf]

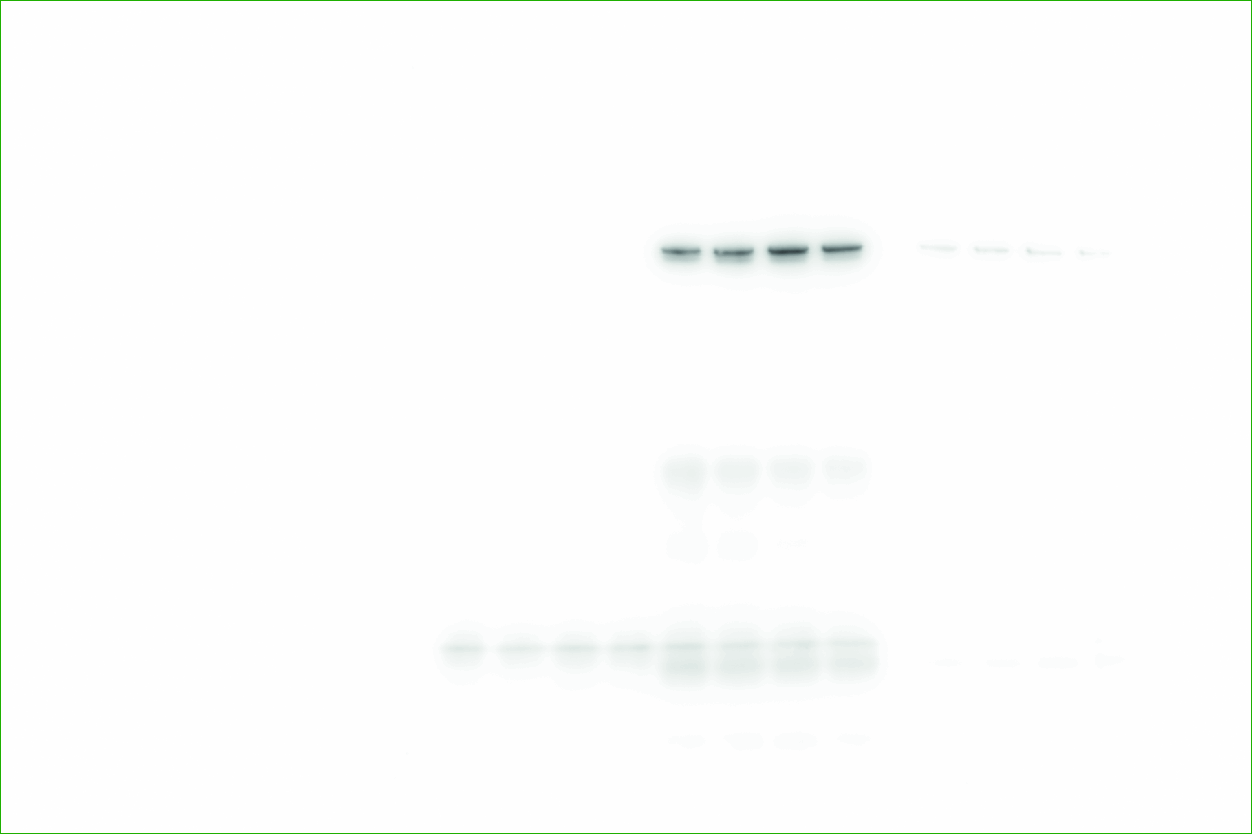

Supplement: Figure 3—source data 1. [file elife-79386-fig3-data1.zip › Figure 3 - source data 1/Figure 3a_source data 4.tif]

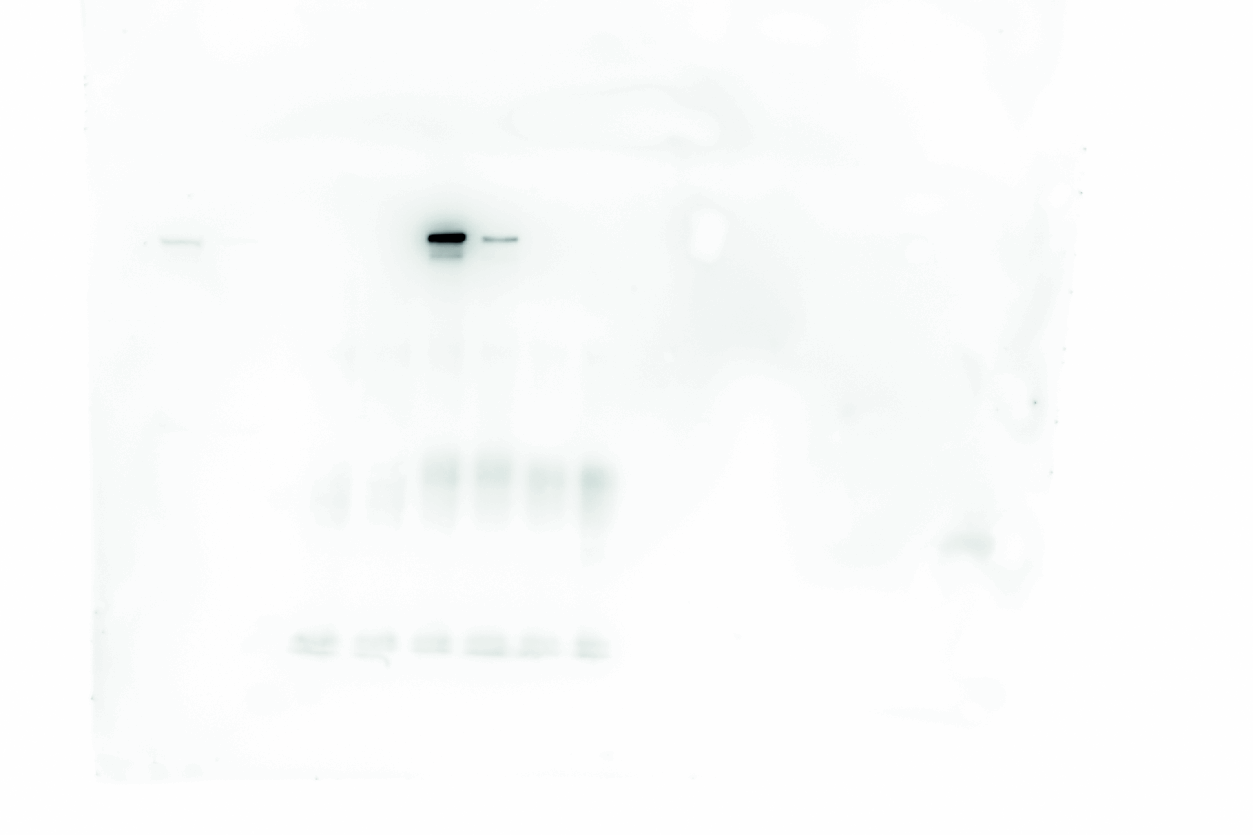

Supplement: Figure 3—source data 1. [file elife-79386-fig3-data1.zip › Figure 3 - source data 1/Figure 3b_source data_2.tif]

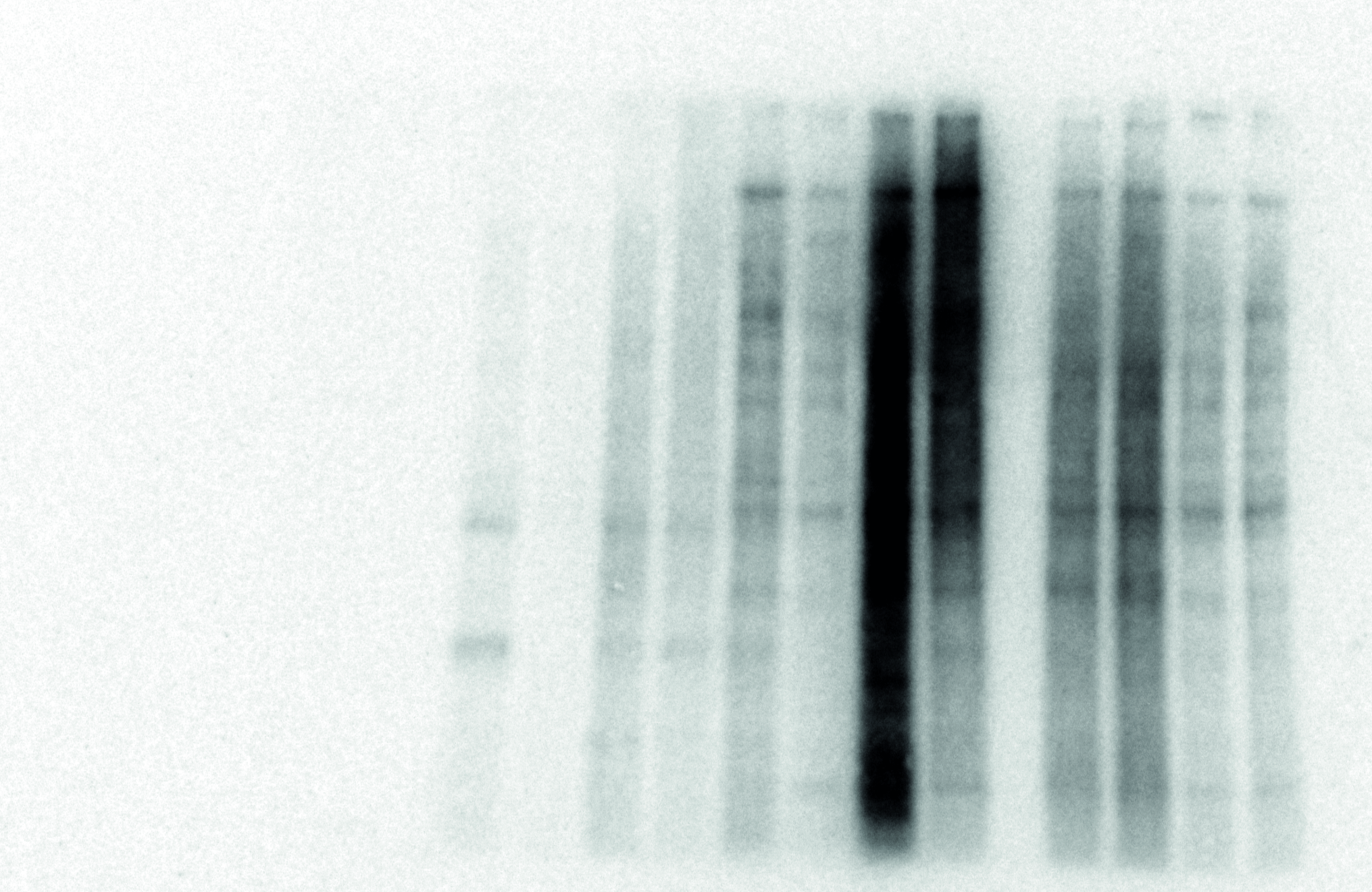

Supplement: Figure 3—source data 1. [file elife-79386-fig3-data1.zip › Figure 3 - source data 1/Figure 3a_source data 1.tif]

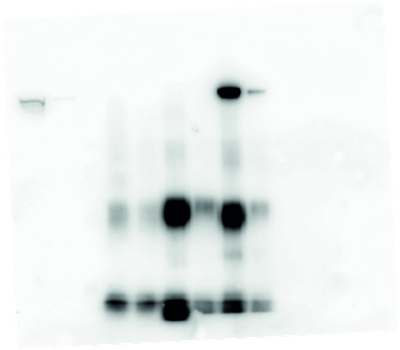

Supplement: Figure 3—source data 1. [file elife-79386-fig3-data1.zip › Figure 3 - source data 1/Figure 3b_source data_3.tif]

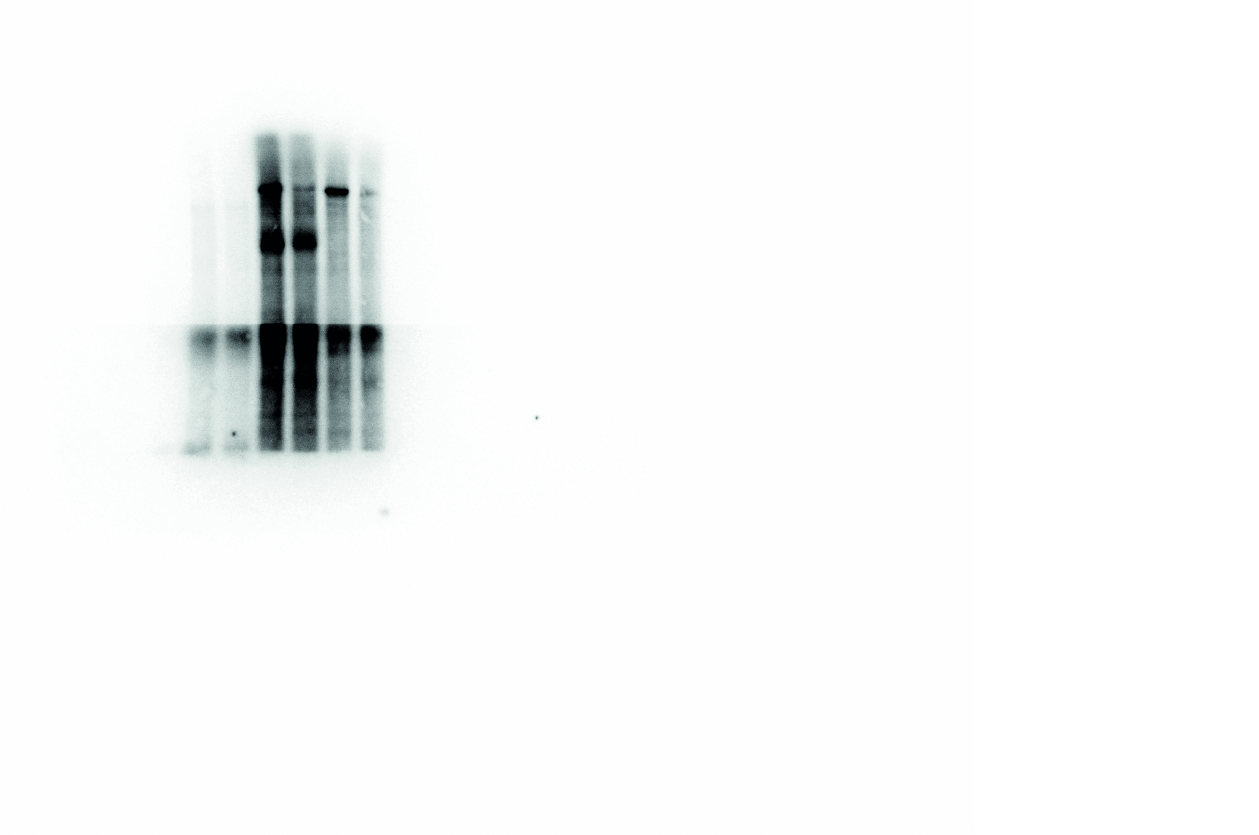

Supplement: Figure 3—source data 1. [file elife-79386-fig3-data1.zip › Figure 3 - source data 1/Figure 3b_source data_1.tif]

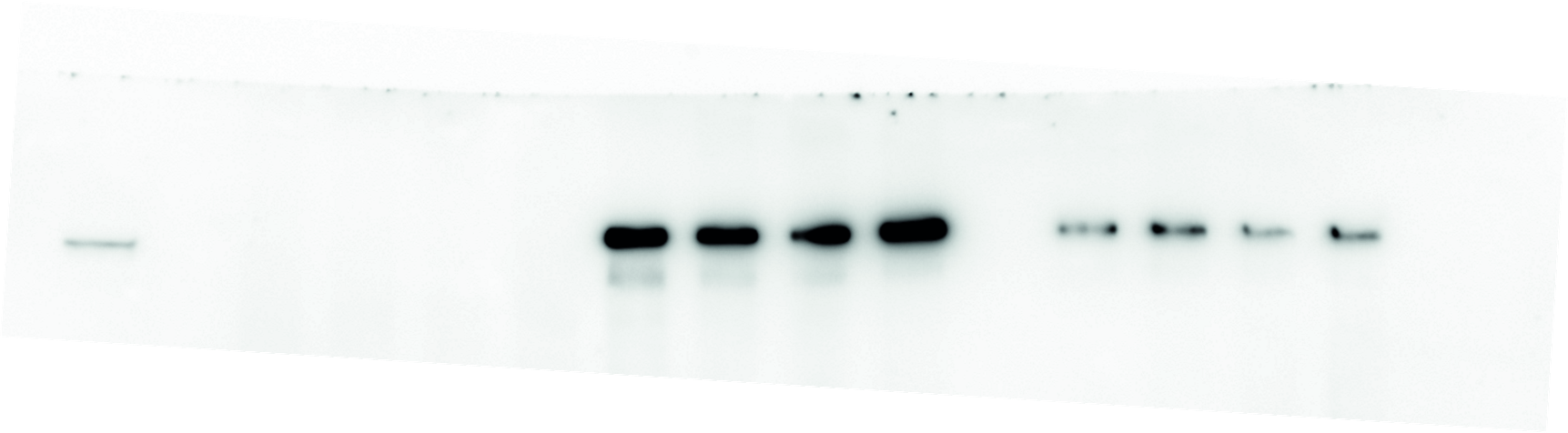

Supplement: Figure 3—source data 1. [file elife-79386-fig3-data1.zip › Figure 3 - source data 1/Figure 3a_source data 2.tif]

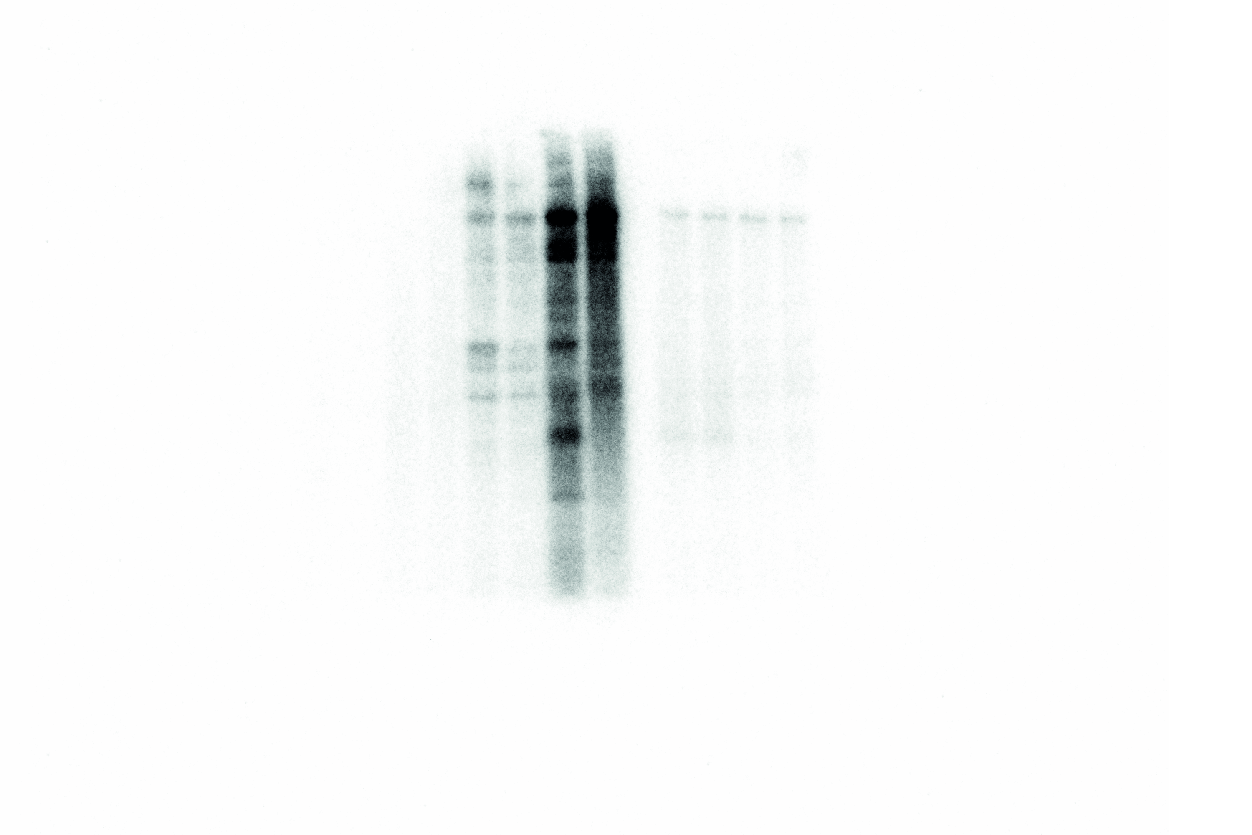

Supplement: Figure 3—source data 1. [file elife-79386-fig3-data1.zip › Figure 3 - source data 1/Figure 3a_source data 3.tif]

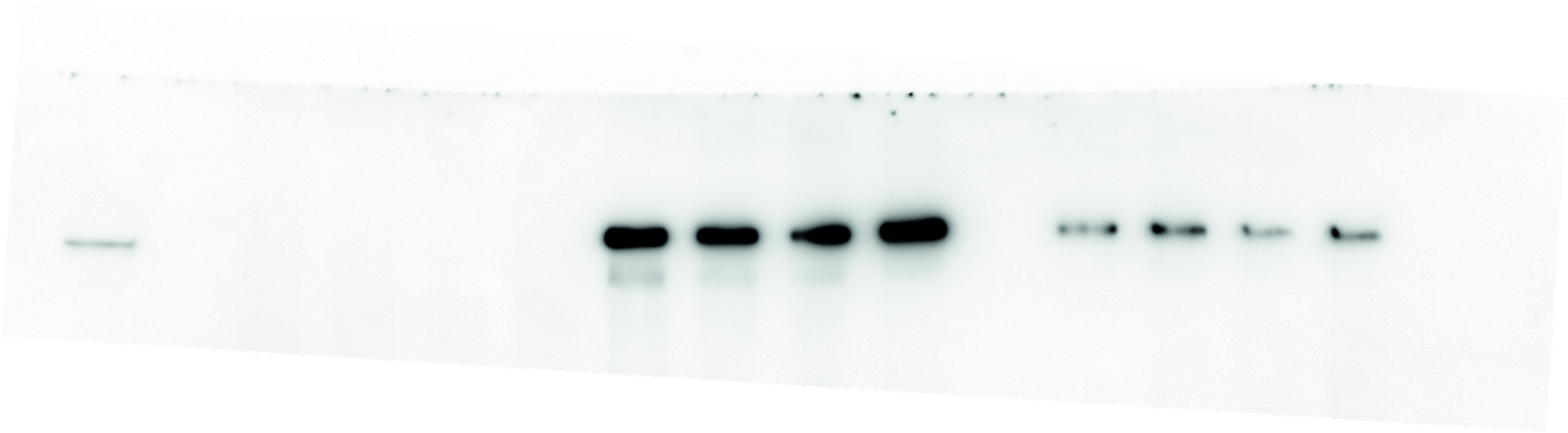

Supplement: Figure 3—figure supplement 1—source data 1. [file elife-79386-fig3-figsupp1-data1.zip › Figure 3 - figure supplement 1 - source data 1/Figure S3a_source data 2.tif]

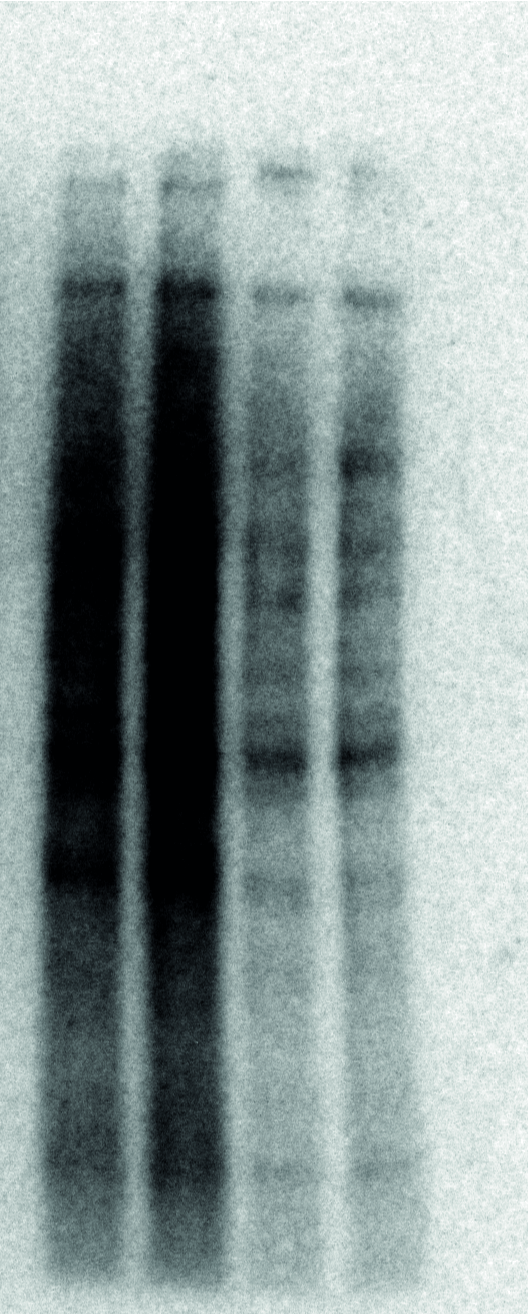

Supplement: Figure 3—figure supplement 1—source data 1. [file elife-79386-fig3-figsupp1-data1.zip › Figure 3 - figure supplement 1 - source data 1/Figure S3a_source data 1.tif]

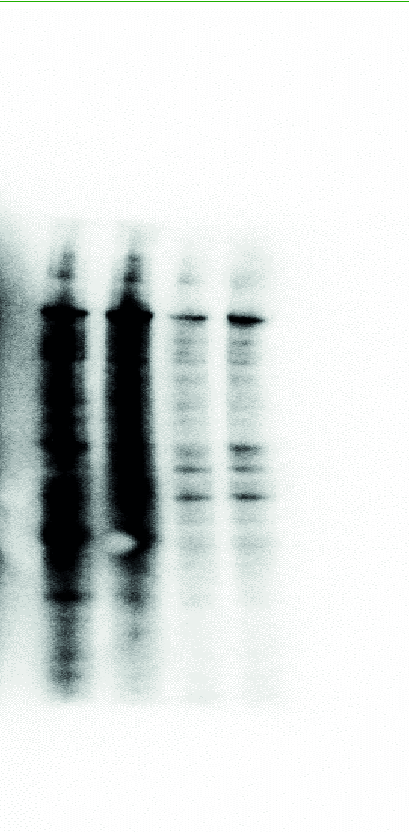

Supplement: Figure 3—figure supplement 1—source data 1. [file elife-79386-fig3-figsupp1-data1.zip › Figure 3 - figure supplement 1 - source data 1/Figure S3b_source data 1.tif]

Figure 4d Source Data

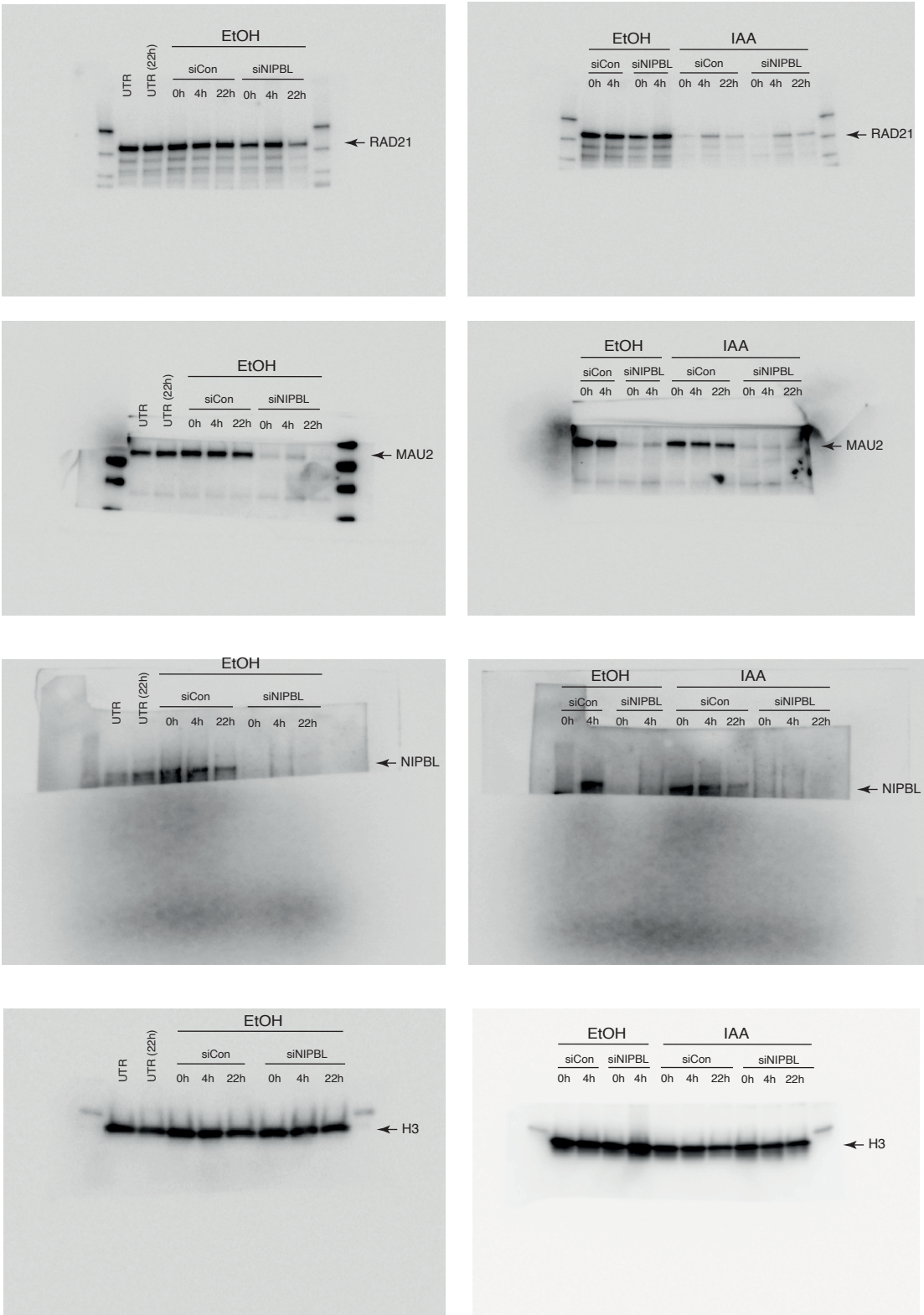

Supplement: Figure 4—source data 1. [file elife-79386-fig4-data1.zip › Figure 4 - source data 1/Source Data_Figure 4.pdf]

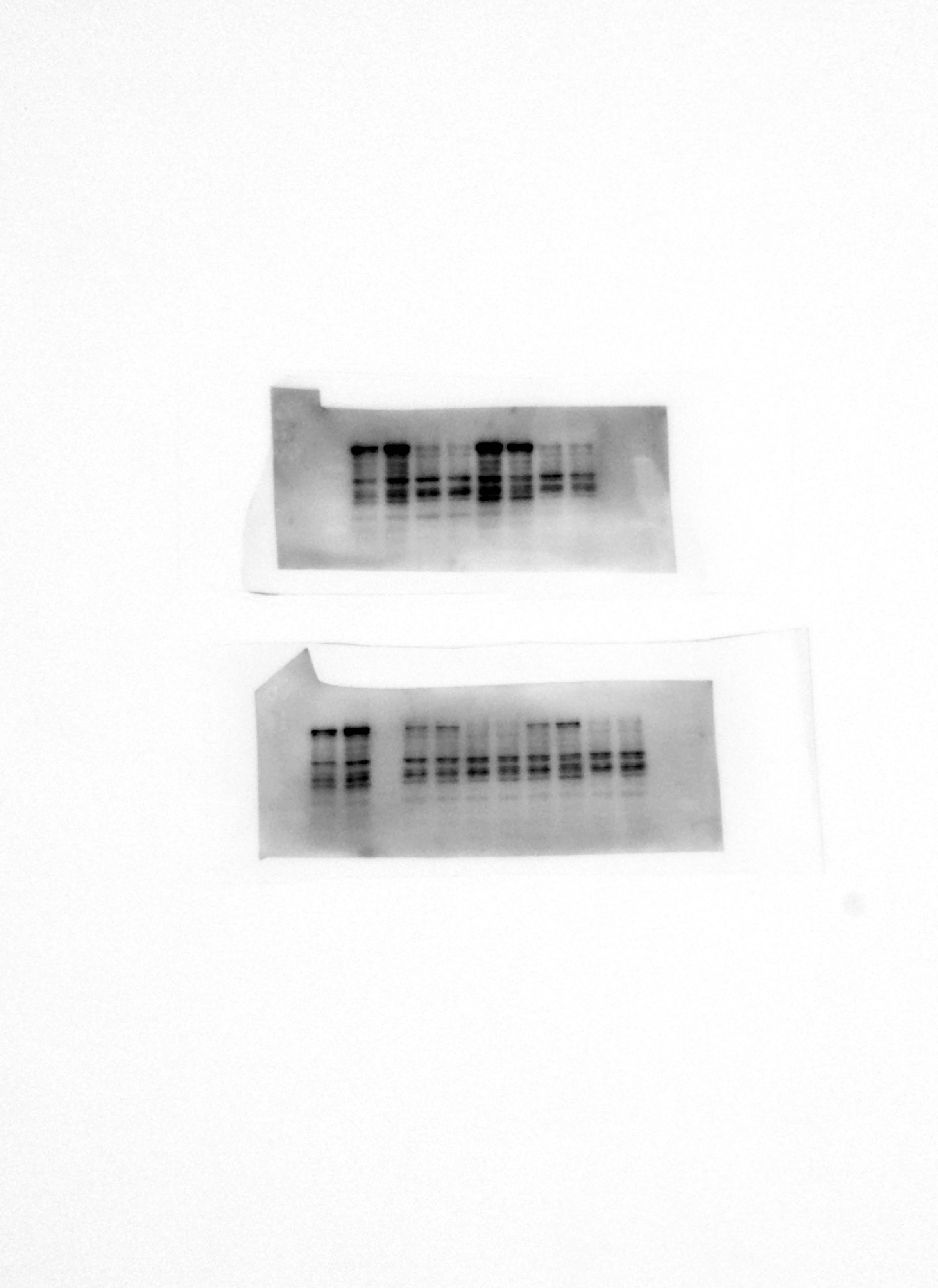

Supplement: Figure 4—figure supplement 1—source data 1. [file elife-79386-fig4-figsupp1-data1.zip › Figure 4 - figure supplement 1 - source data 1/Figure S4e_source data 3.jpg]

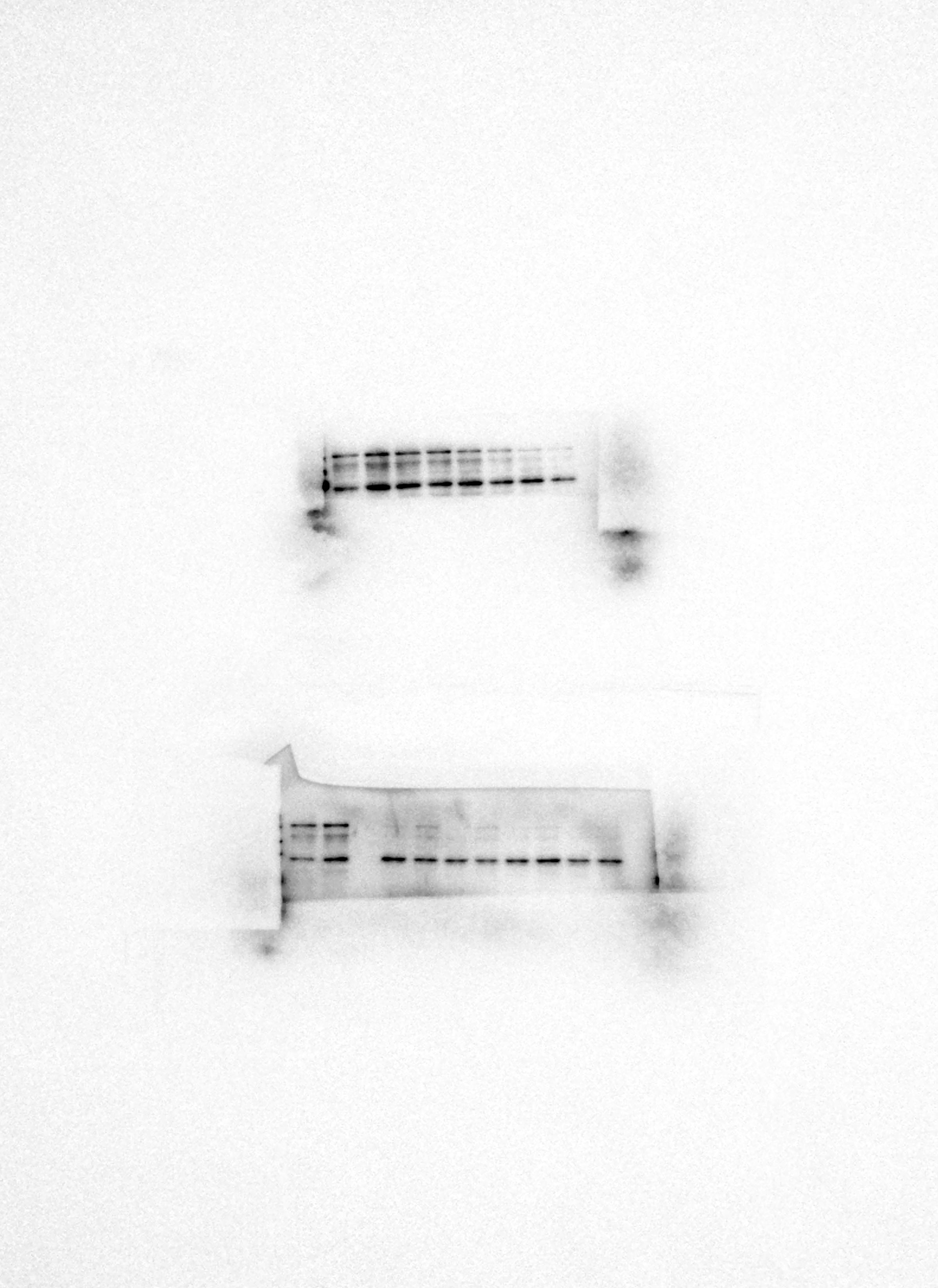

Supplement: Figure 4—figure supplement 1—source data 1. [file elife-79386-fig4-figsupp1-data1.zip › Figure 4 - figure supplement 1 - source data 1/Figure S4e_source data 2.jpg]

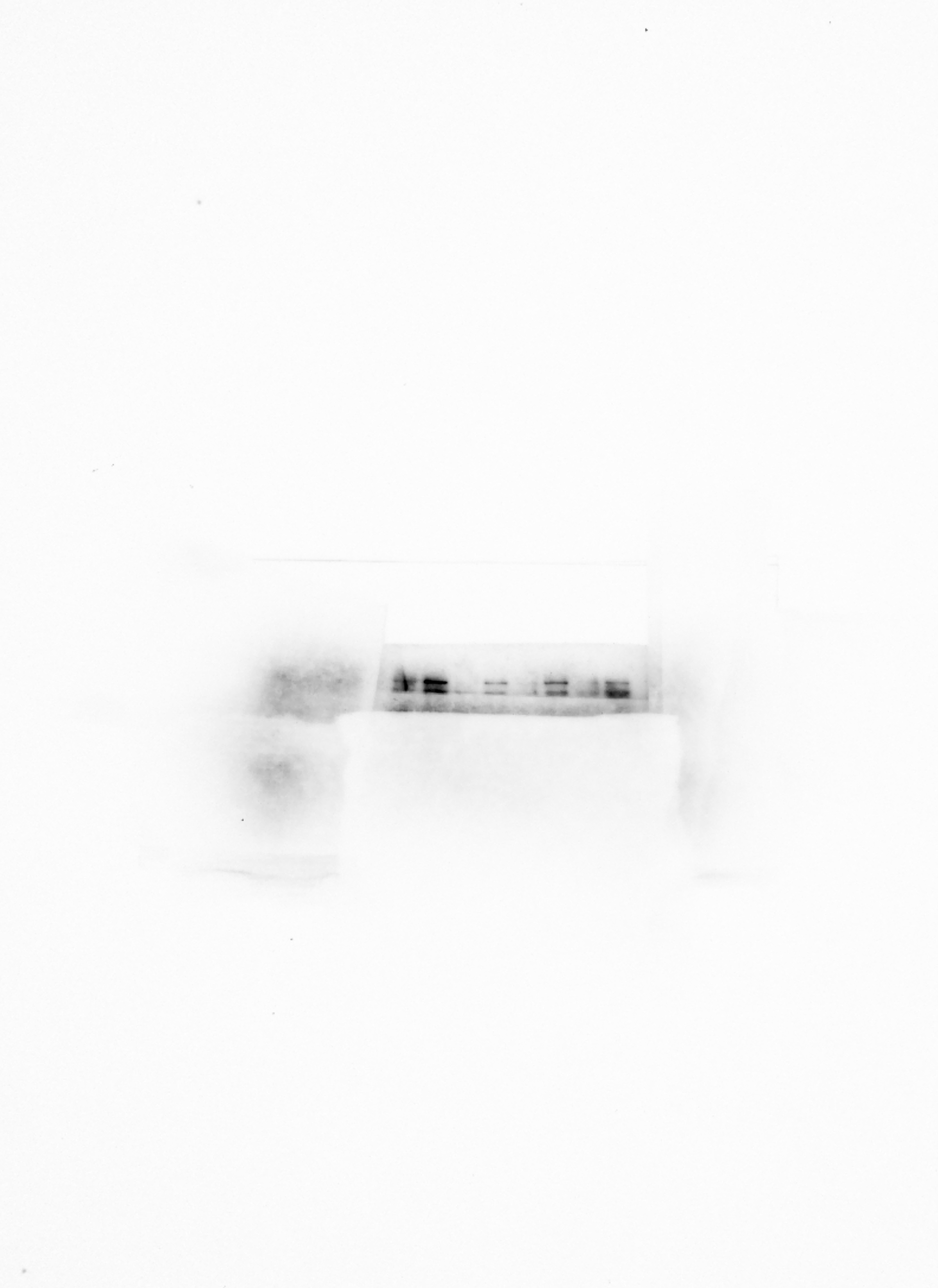

Supplement: Figure 4—figure supplement 1—source data 1. [file elife-79386-fig4-figsupp1-data1.zip › Figure 4 - figure supplement 1 - source data 1/Figure S4e_source data 1.png]

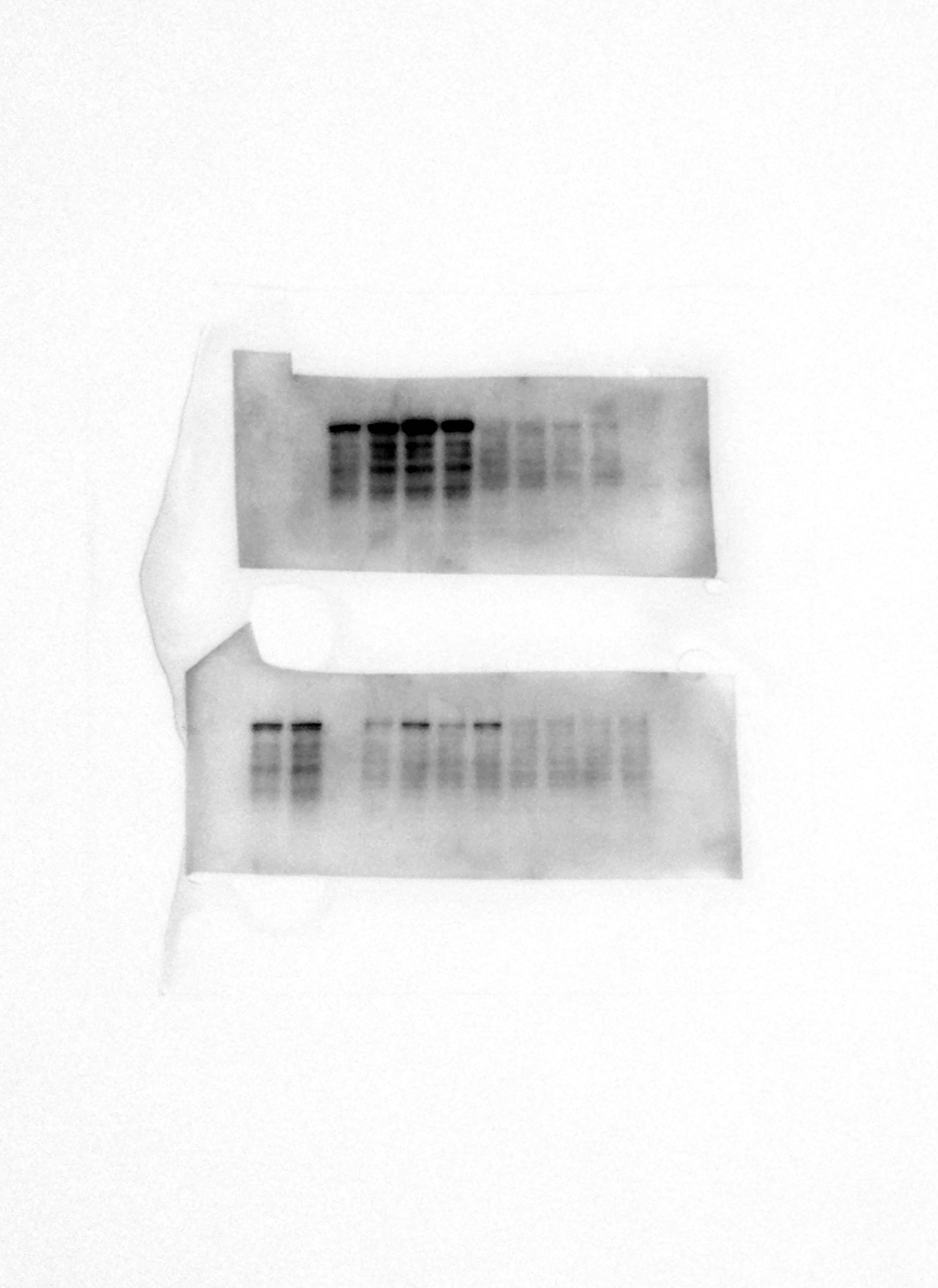

Supplement: Figure 4—figure supplement 1—source data 1. [file elife-79386-fig4-figsupp1-data1.zip › Figure 4 - figure supplement 1 - source data 1/Figure S4e_source data 4.jpg]

Figure 5c Source Data

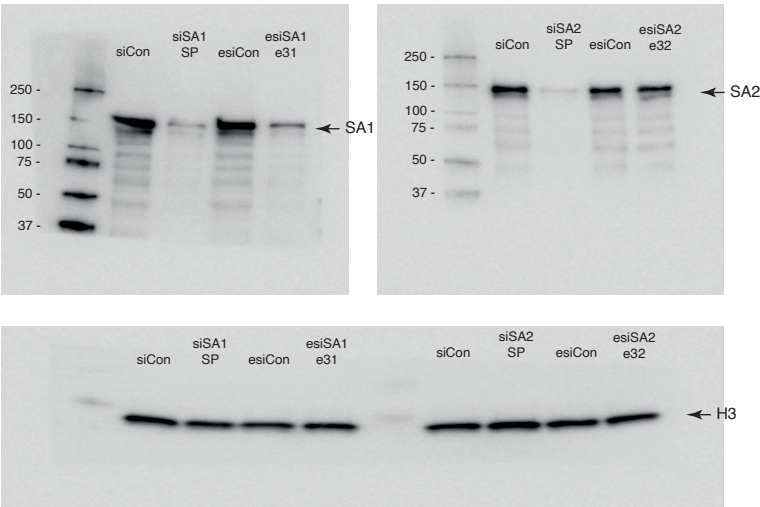

Figure 5d Source Data

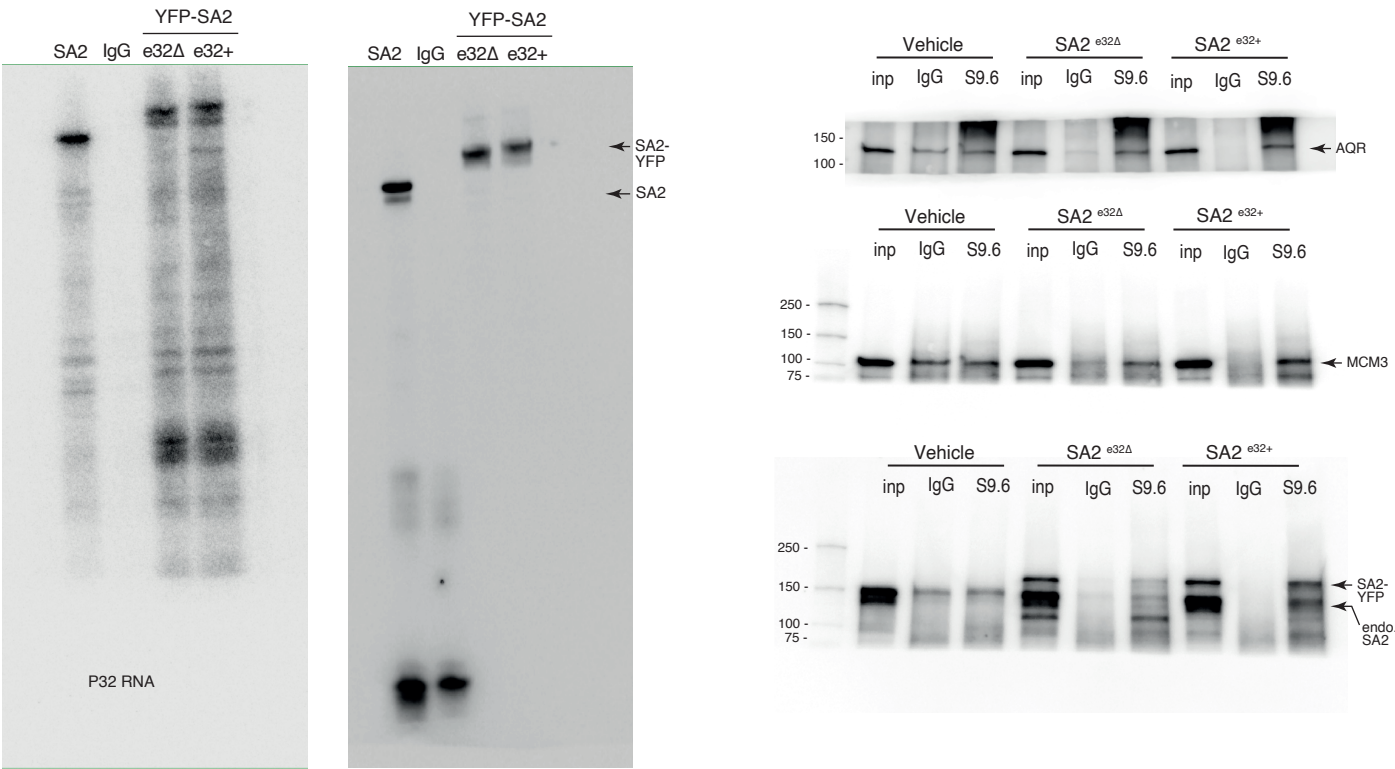

Supplement: Figure 5—source data 1. [file elife-79386-fig5-data1.zip › Figure 5 - source data 1/Source Data_Figure 5.pdf]

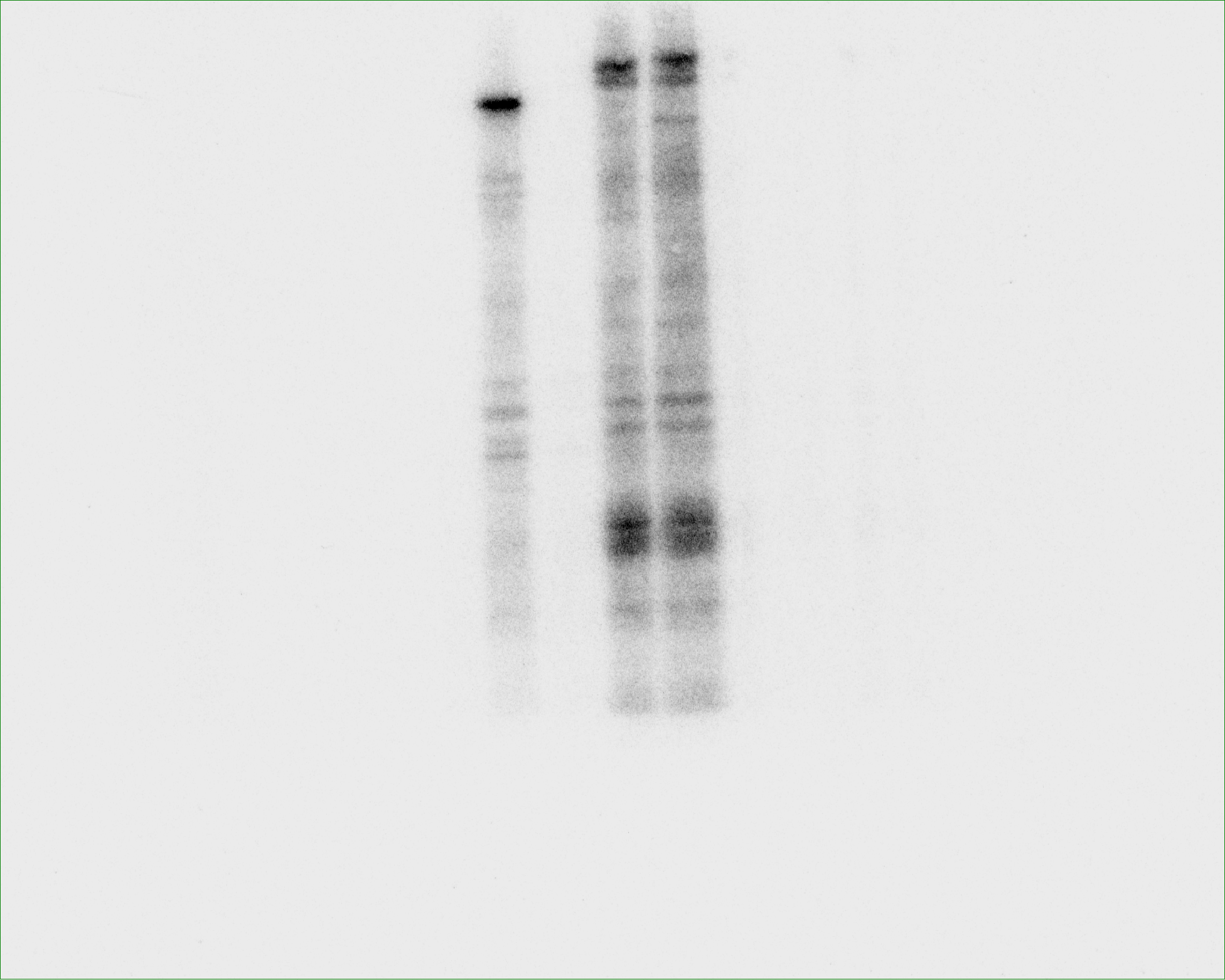

Supplement: Figure 5—source data 1. [file elife-79386-fig5-data1.zip › Figure 5 - source data 1/Figure 5d_source data 1.tif]

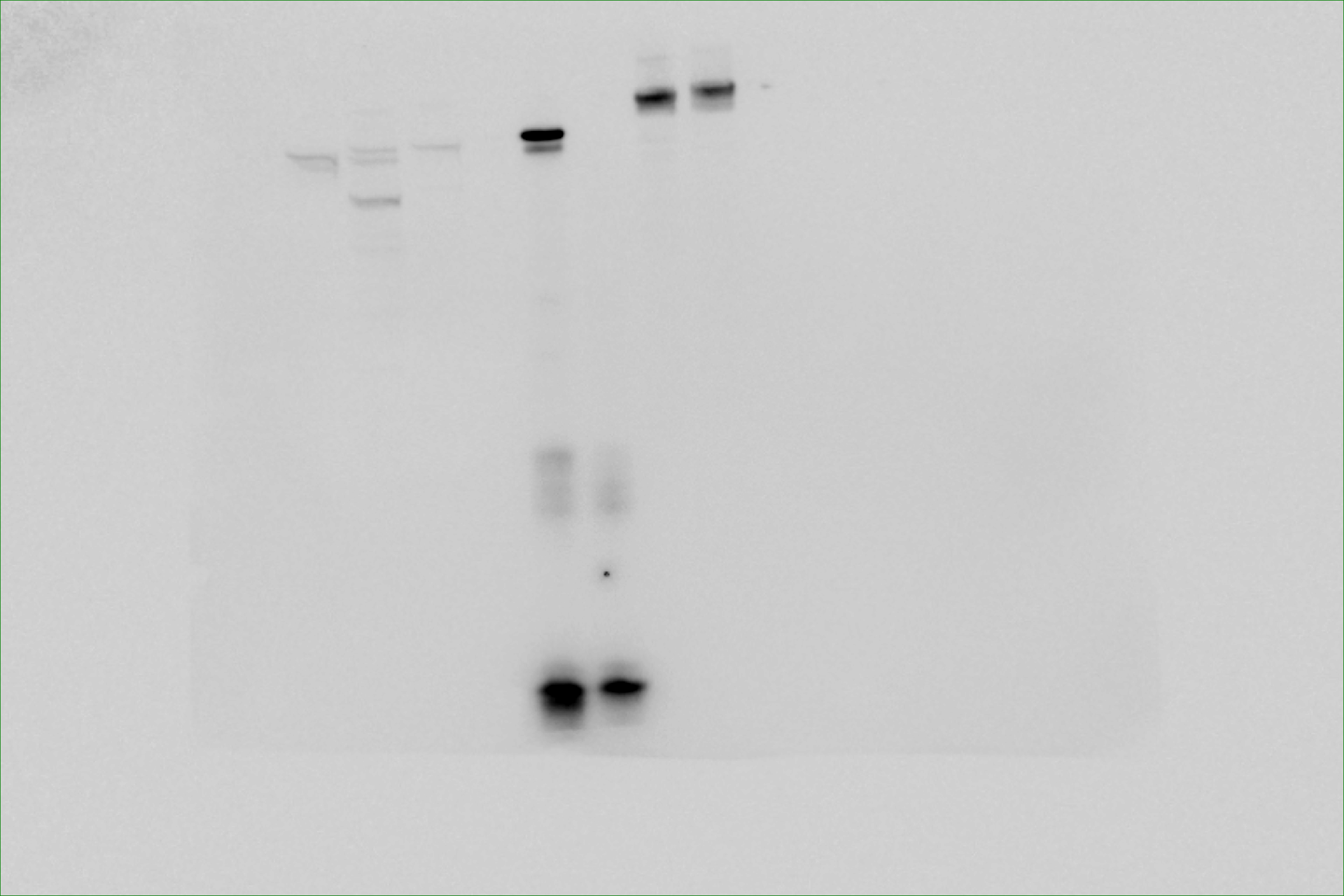

Supplement: Figure 5—source data 1. [file elife-79386-fig5-data1.zip › Figure 5 - source data 1/Figure 5d_source data 2.tif]

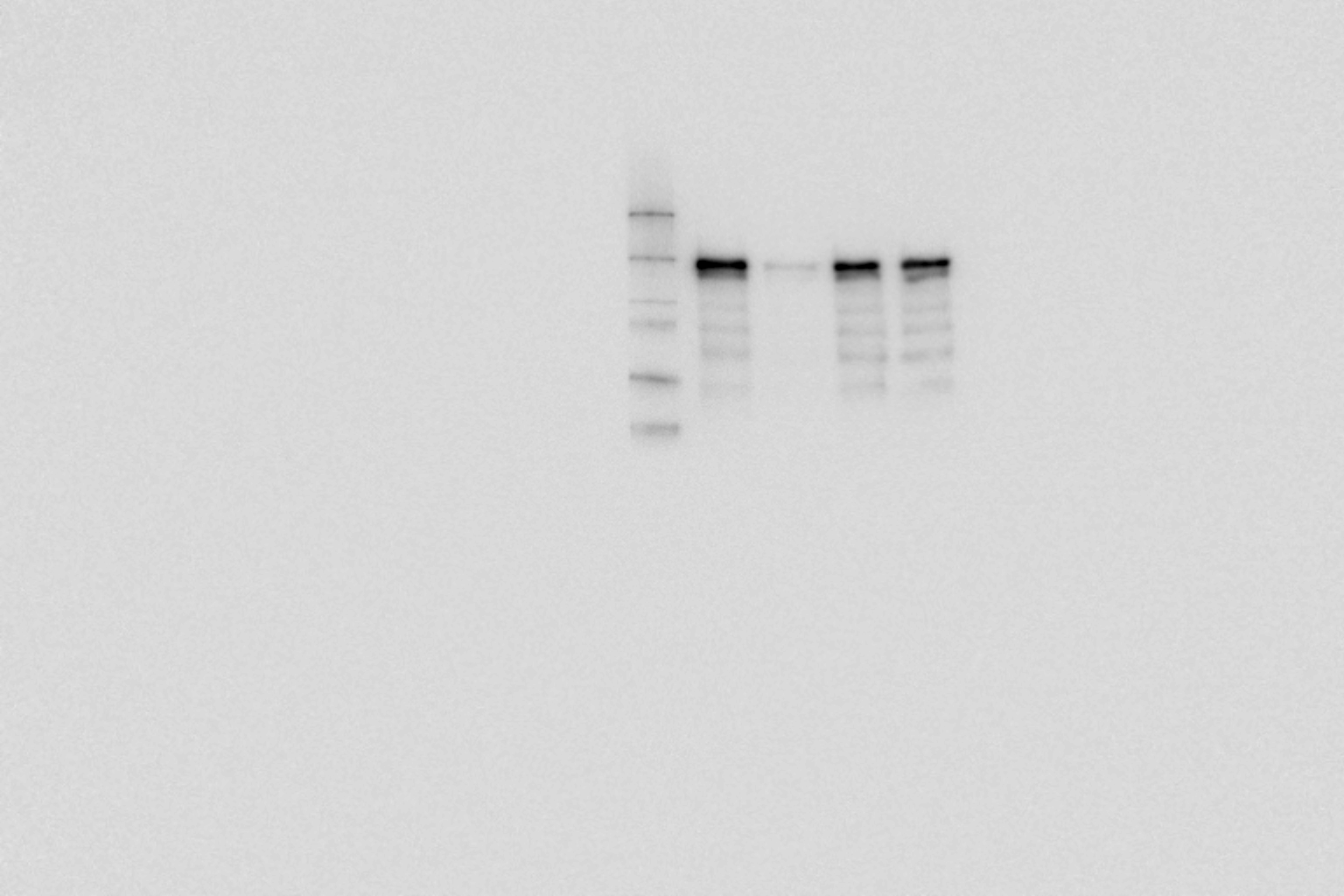

Supplement: Figure 5—source data 1. [file elife-79386-fig5-data1.zip › Figure 5 - source data 1/Figure 5c_source data 2.tif]

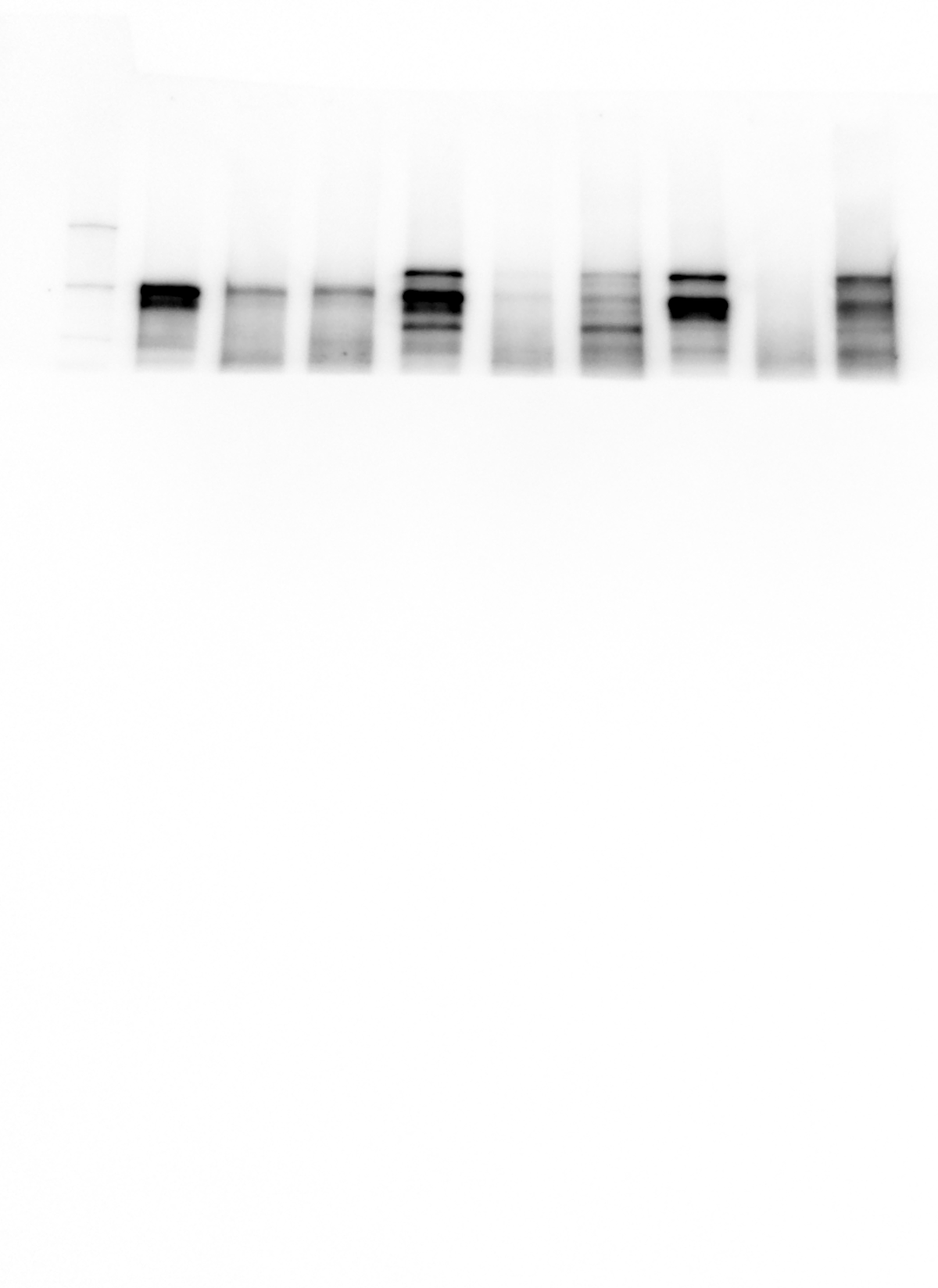

Supplement: Figure 5—source data 1. [file elife-79386-fig5-data1.zip › Figure 5 - source data 1/Figure 5f_source data_3.png]

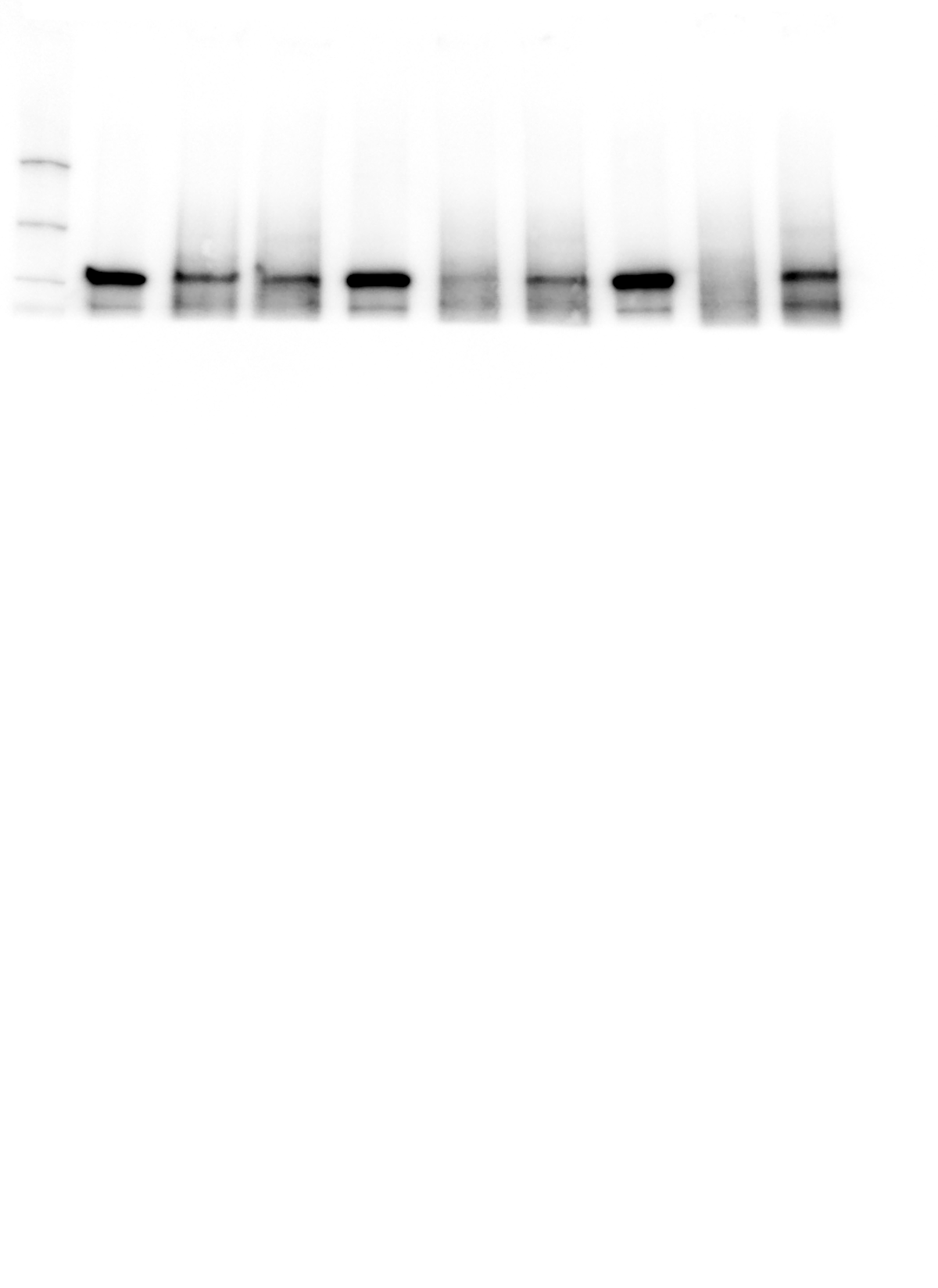

Supplement: Figure 5—source data 1. [file elife-79386-fig5-data1.zip › Figure 5 - source data 1/Figure 5f_source data_2.png]

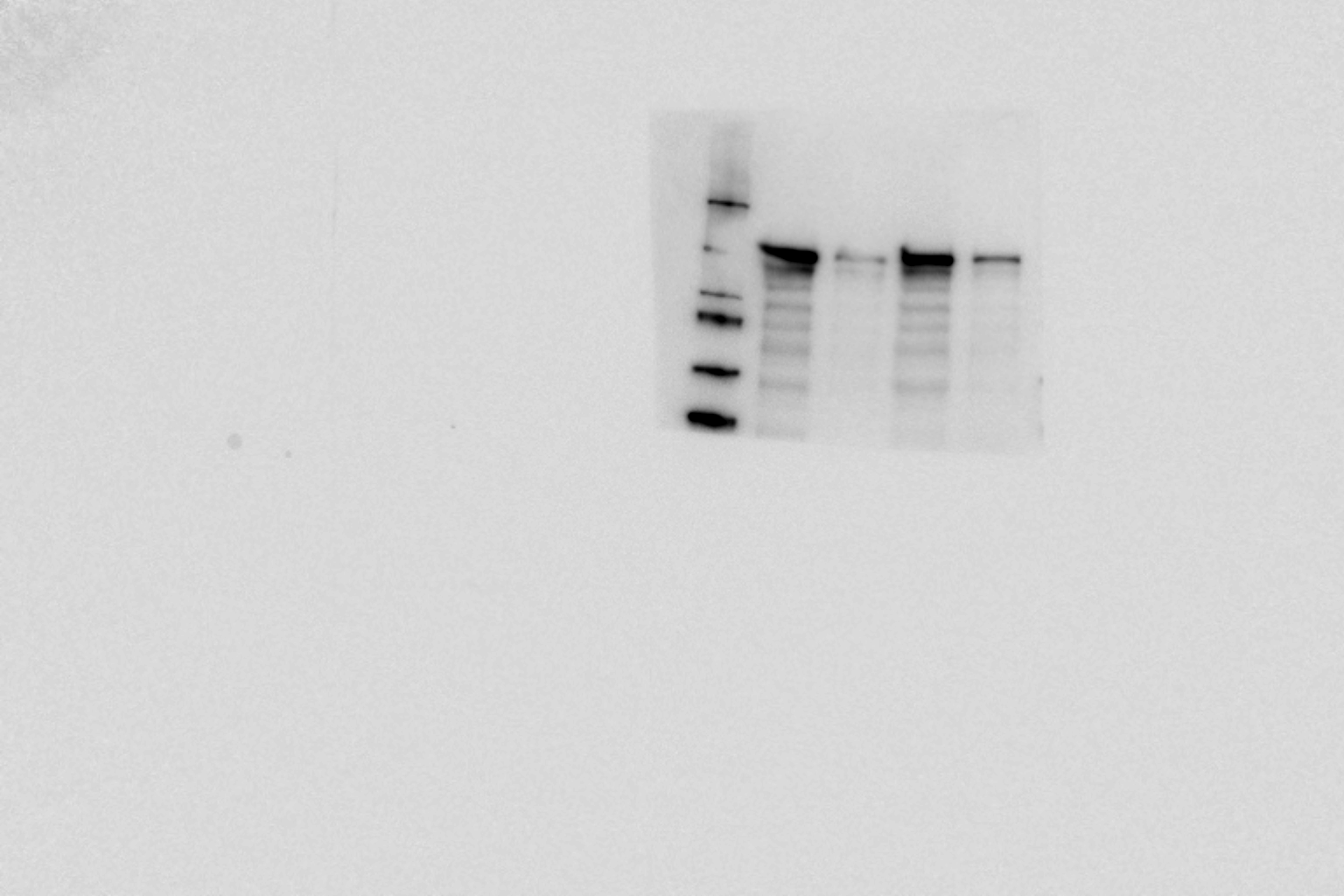

Supplement: Figure 5—source data 1. [file elife-79386-fig5-data1.zip › Figure 5 - source data 1/Figure 5c_source data 1.tif]

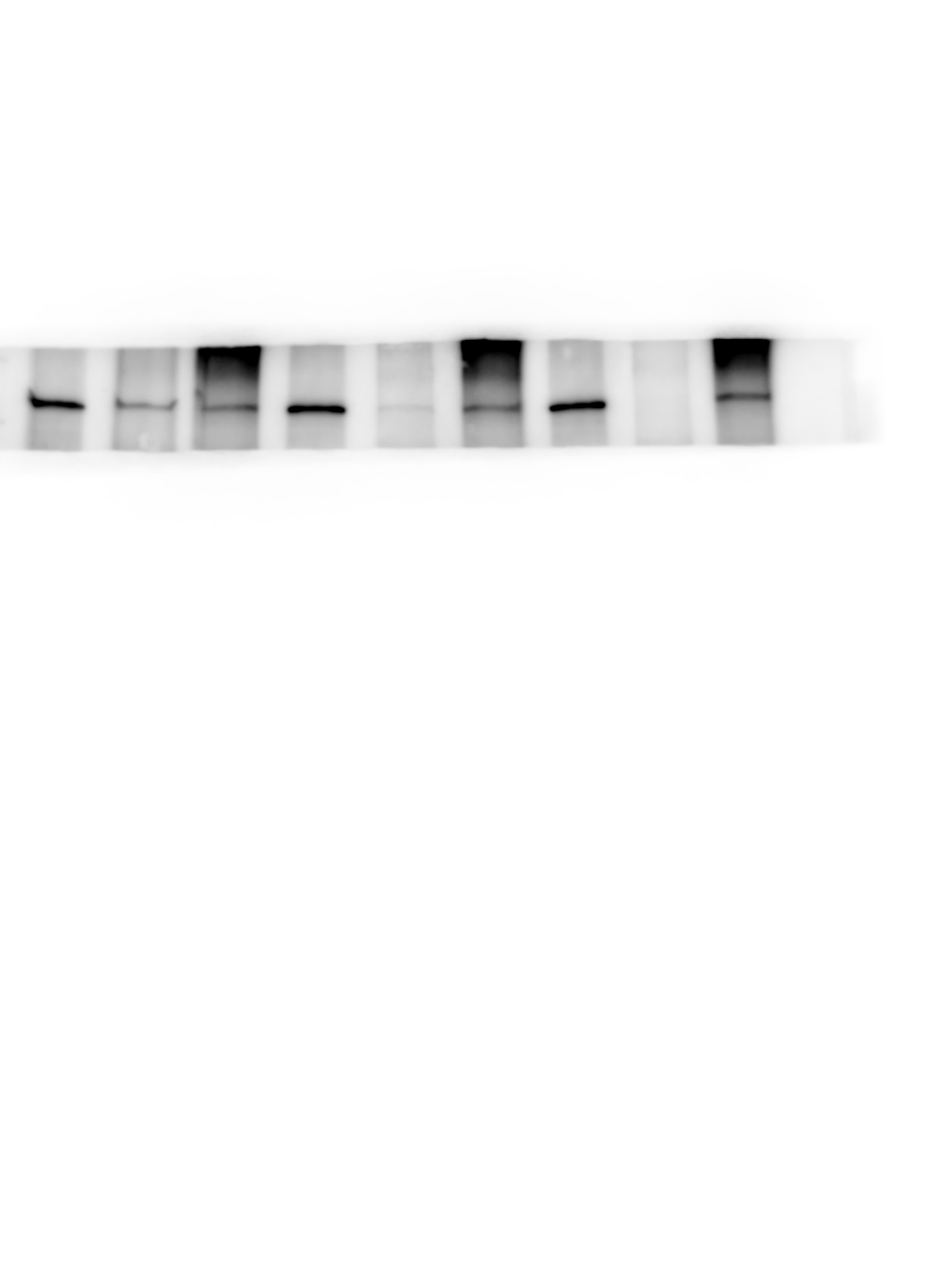

Supplement: Figure 5—source data 1. [file elife-79386-fig5-data1.zip › Figure 5 - source data 1/Figure 5f_source data_1.png]
