# Supplementary material for: Cohesin-independent STAG proteins interact with RNA and R-loops and promote complex loading
Source: eLife. 2023 Apr 3;12:e79386. doi: 10.7554/eLife.79386 (PMC10238091; doi:10.7554/eLife.79386)
Supplement: Figure 1—figure supplement 1—source data 1. [file elife-79386-fig1-figsupp1-data1.zip › Figure 1 - figure supplement 1 - source data 1/Source Data_SUPP Figure 1.pdf]

Source Data for Figure 1 - figure supplement 1

Porter, Li et al.

Figure S1a Source Data.

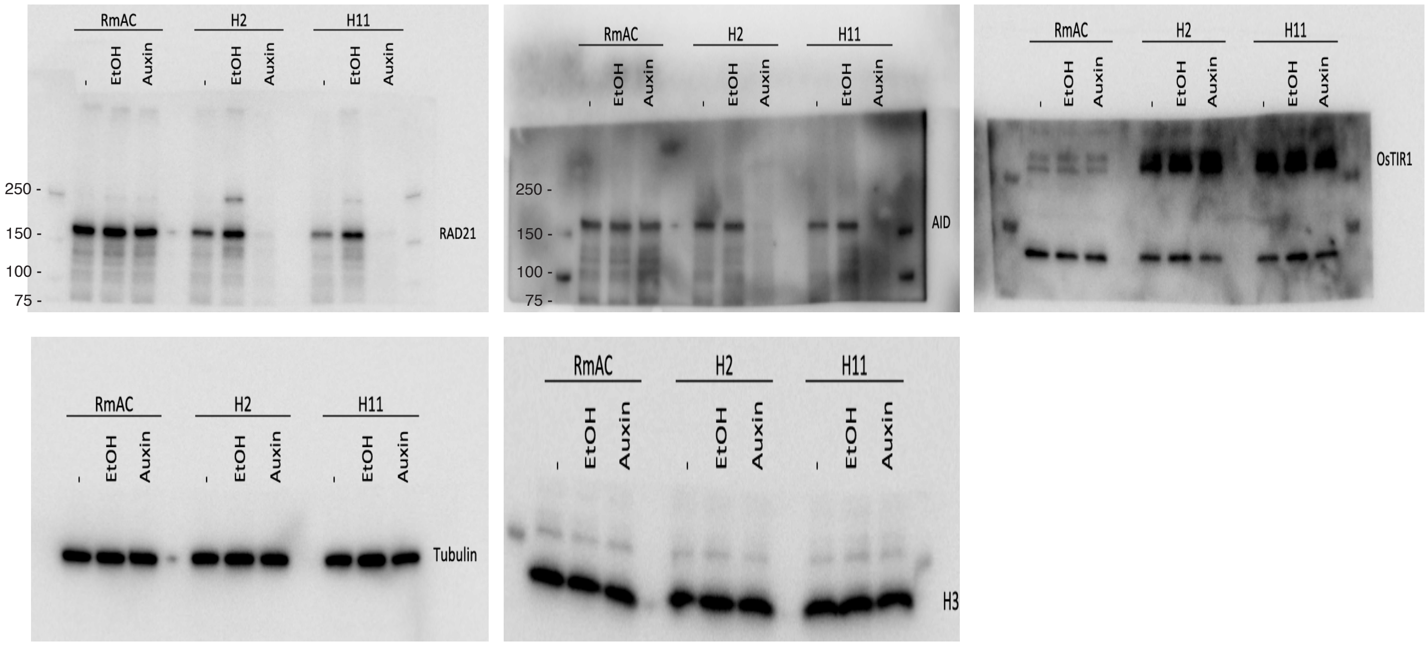

Figure S1c Source Data.

These were among the earliest experiments done and we are unable to find the uncropped blot images for this data.
