# Supplementary material for: Cohesin-independent STAG proteins interact with RNA and R-loops and promote complex loading
Source: eLife. 2023 Apr 3;12:e79386. doi: 10.7554/eLife.79386 (PMC10238091; doi:10.7554/eLife.79386)

Source Data for Figure 3 - figure supplement 1

Porter, Li et al.

Figure S3a -e, full blots for CLIP experiments are already shown in the Supplementary file.

Figure S3h source data.

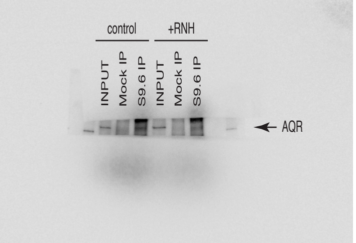

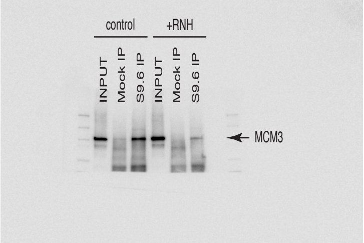

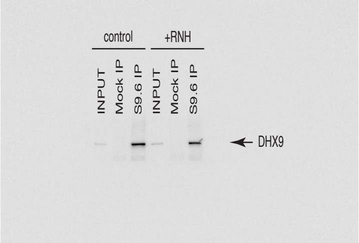

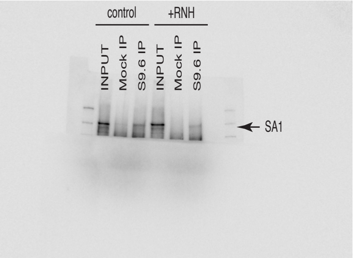

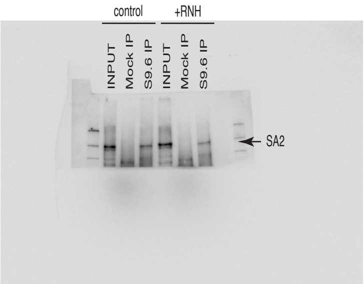

Supplement: Figure 3—figure supplement 1—source data 1. [file elife-79386-fig3-figsupp1-data1.zip › Figure 3 - figure supplement 1 - source data 1/Source Data_SUPP Figure 3_v2.pdf]
