# Supplementary material for: Cohesin-independent STAG proteins interact with RNA and R-loops and promote complex loading
Source: eLife. 2023 Apr 3;12:e79386. doi: 10.7554/eLife.79386 (PMC10238091; doi:10.7554/eLife.79386)

Figure 4f Source Data

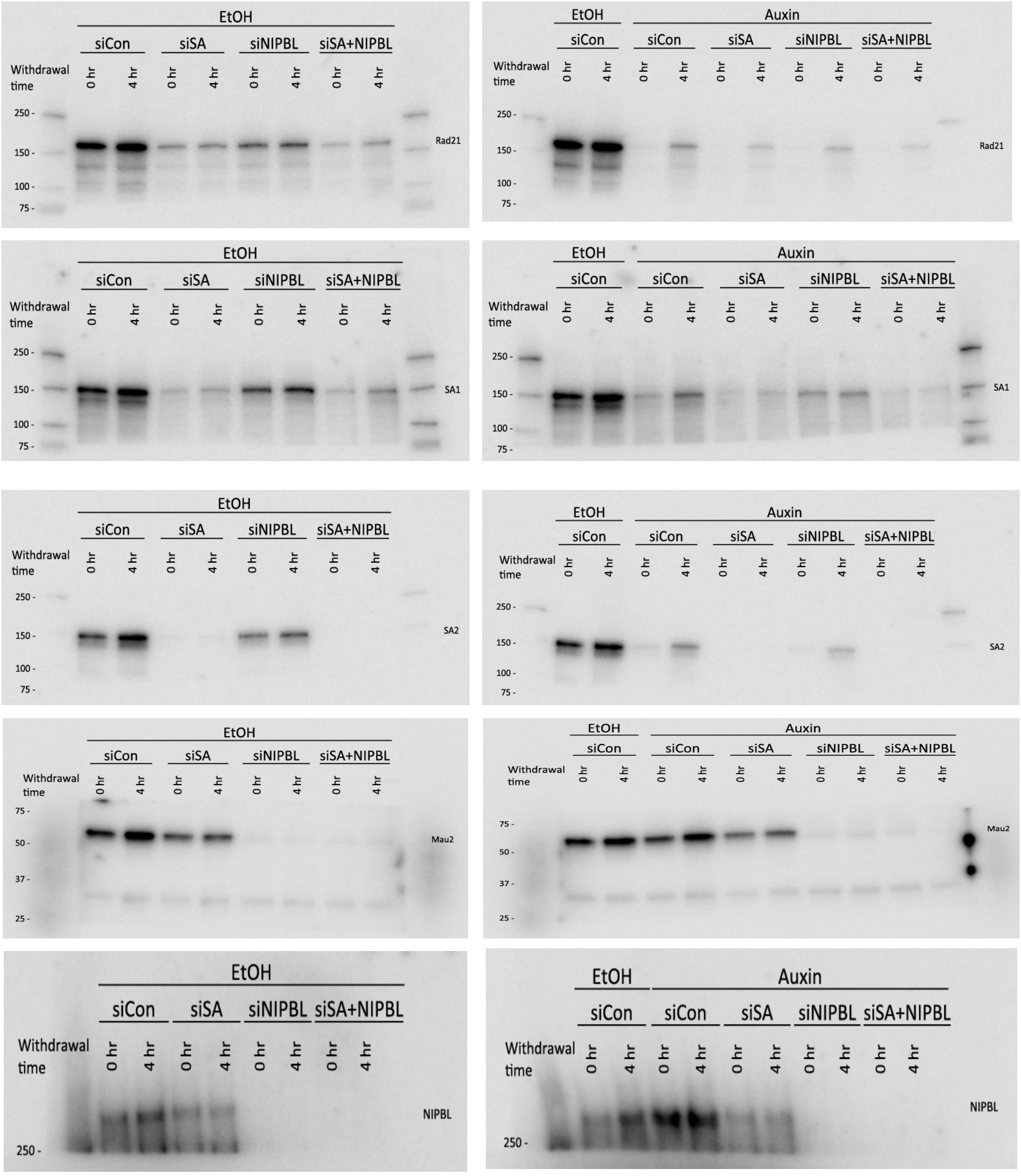

Supplement: Figure 4—source data 1. [file elife-79386-fig4-data1.zip › Figure 4 - source data 1/Source Data_Figure 4_page2.pdf]
