# Supplementary material for: Cohesin-independent STAG proteins interact with RNA and R-loops and promote complex loading
Source: eLife. 2023 Apr 3;12:e79386. doi: 10.7554/eLife.79386 (PMC10238091; doi:10.7554/eLife.79386)

Source Data for Figure 4 - figure Supplement 1

Porter, Li et al.

Figure S4c source data is the same as the data shown in Figure 4d and its source file.

Figure S4e source data (layout is the same as shown in S4e).

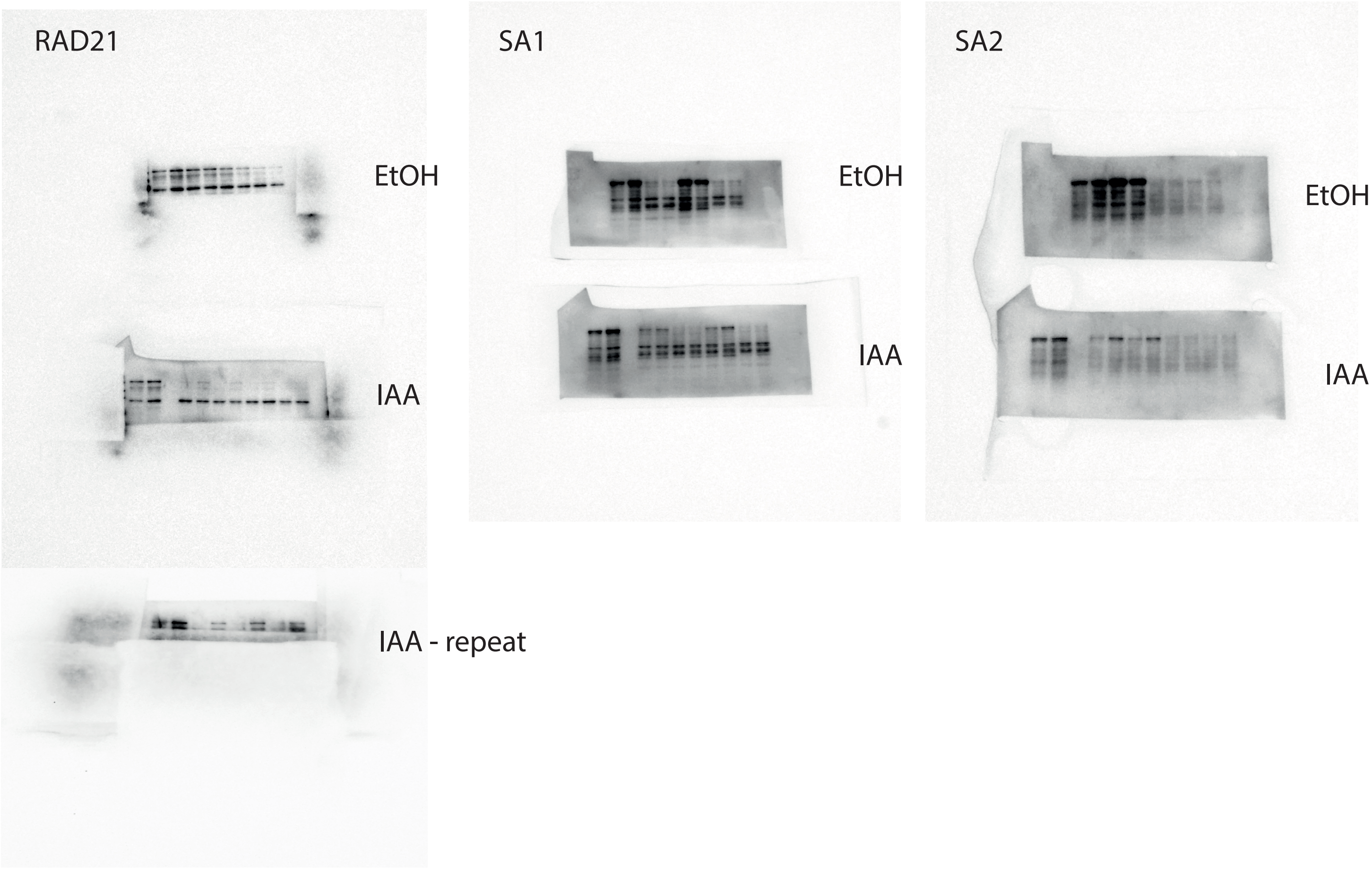

Supplement: Figure 4—figure supplement 1—source data 1. [file elife-79386-fig4-figsupp1-data1.zip › Figure 4 - figure supplement 1 - source data 1/Source Data_SUPP Figure 4.pdf]
